# Hydrogen-Rich Gas Production from Municipal Solid Waste via Integrated Pyrolysis and Catalytic Steam Reforming over Ni/Al_2_O_3_ Catalyst

**DOI:** 10.3390/molecules31162917

**Published:** 2026-08-20

**Authors:** Ivan Pedro Lazzarotto, Oscar de Almeida Neuwald, Lucas David Biondo, Daniele Perondi, Christian Manera, Marcelo Godinho

**Affiliations:** 1 Engineering of Processes and Technologies Post-Graduate Program, University of Caxias do Sul—UCS, Caxias do Sul 95070-560, Braziloaneuwald@ucs.br (O.d.A.N.); lucas.biondo@gmail.com (L.D.B.); dperondi1@ucs.br (D.P.); cmanera1@ucs.br (C.M.); 2 UCSGRAPHENE Research and Development Unit, University of Caxias do Sul—UCS, Caxias do Sul 95070-560, Brazil

**Keywords:** municipal solid waste, pyrolysis, steam catalytic reforming, hydrogen

## Abstract

The transition to a hydrogen-based economy requires efficient and sustainable technologies to convert waste into clean energy carriers. This study investigates the production of hydrogen-rich gas through the integrated pyrolysis steam reforming (PSR) and integrated pyrolysis (PYR) and catalytic steam reforming (PCSR) of real municipal solid waste (MSW). PCSR experiments were conducted in a two-stage series reactor system: an initial pyrolysis stage at 500 °C followed by a catalytic steam reforming stage at 900 °C over a commercial Ni/Al_2_O_3_ catalyst (9.8 wt.% Ni). Three real MSW samples from the Serra Gaúcha region (Brazil) were evaluated: organic-rich (A), polymeric-rich (B), and a mixed real collection fraction (C). Gas yields from PYR to PCSR increased from 0.44 to 1.18 Nm^3^·kgMSW^−1^, from 0.66 to 1.54 Nm^3^·kgMSW^−1^, and from 0.35 to 1.50 Nm^3^·kgMSW^−1^ for (A), (B), and (C) samples, respectively. Hydrogen concentrations of PCSR were between 32% and 39% volume for all samples, with a marked reduction in CH_4_ and CO levels due to the promotion of water–gas shift and methane reforming reactions over the nickel active sites. The PCSR process using a Ni/Al_2_O_3_ catalyst proves to be a highly effective route for maximizing hydrogen production from real MSW, offering a robust technological solution for energy valorization and carbon footprint reduction.

## 1. Introduction

The exponential growth of the global population, coupled with rapid urbanization and shifting consumption patterns, has led to an unprecedented increase in the generation of municipal solid waste (MSW). Current estimates indicate that global waste production exceeds 2.01 billion tons annually, a figure projected to rise by 70% by 2050 if business-as-usual scenarios persist [1]. In the Brazilian context, the management of these residues remains a critical challenge despite the guidelines established by the National Solid Waste Policy (PNRS) [2]. The PNRS prioritizes the non-generation, reduction, reuse, and recycling of waste, yet a significant portion of MSW is still disposed of in landfills or controlled dumps, resulting in severe environmental liabilities, including greenhouse gas emissions and leachate contamination. Consequently, there is an urgent need for advanced thermochemical technologies capable of diverting waste from landfills while recovering its inherent energetic and chemical value.

Simultaneously, the global energy matrix is undergoing a profound transition toward decarbonization to mitigate the effects of climate change. Hydrogen (H_2_) has emerged as a pivotal clean energy vector due to its high energy density and carbon-free combustion, making it essential for the “hard-to-abate” industrial sectors and heavy-duty transportation [3,4]. In Brazil, the National Hydrogen Program (PNH2) aims to consolidate the country as a global leader in low-carbon hydrogen production, leveraging its diverse biomass and waste resources [5]. However, the sustainability of the hydrogen economy depends heavily on the development of “green” or “circular” production pathways that do not rely on fossil fuel steam methane reforming (SMR) [6]. In this regard, the thermochemical conversion of MSW via pyrolysis and subsequent catalytic steam reforming presents a promising route for decentralized hydrogen production [7,8,9,10,11,12].

Pyrolysis (PYR) is a thermochemical process in which organic matter is degraded in the total or near-total absence of oxygen, converting the complex polymeric structures of municipal solid waste (MSW) into biochar, condensable bio-oil, and non-condensable gases [13,14,15].

Pyrolysis steam reforming (PSR) is a two-stage thermochemical process in which the volatile products generated during the pyrolysis of biomass or municipal solid waste (MSW) are directly fed into a steam reforming reactor operating at high temperatures in the presence of steam. Unlike catalytic configurations, PSR relies primarily on thermal steam reforming and cracking reactions to convert hydrocarbons, oxygenated compounds, and tar into a hydrogen-rich syngas. The process promotes higher hydrogen production and lower tar content than conventional PYR, although its efficiency is strongly dependent on reforming temperature, steam-to-carbon (S/C) ratio, residence time, and feedstock composition. Because no catalyst is employed, catalyst deactivation is avoided, but higher temperatures are generally required to achieve high conversion efficiencies.

While pyrolysis alone yields a gas fraction with moderate heating value, the integration of an in-line catalytic steam reforming stage—often referred to as the pyrolysis and catalytic steam reforming in series (PCSR) process—significantly enhances hydrogen yields by cracking heavy hydrocarbons and tars [7,8,9,10,11,12]. The PCSR process has been considered attractive for the production of hydrogen-rich gas (H_2_) [16,17]. For instance, the use of self-derived char-based catalysts has shown potential in reducing the costs associated with tar removal while promoting the water–gas shift reaction [18]. Furthermore, the optimization of operating parameters such as temperature and steam-to-carbon (S/C) ratios is crucial for maximizing H_2_ selectivity and preventing catalyst deactivation by coking [19].

Despite the technological advancements, a significant gap remains in the literature regarding the use of real, heterogeneous MSW. Most existing research utilizes synthetic mixtures or “model” compounds (such as pure cellulose or specific plastics) to ensure reproducibility [16,17,20,21,22]. However, real MSW is characterized by extreme physical and chemical heterogeneity, containing varying proportions of food waste, paper, plastics, textiles, and inert materials, which vary significantly by geographic location and socioeconomic factors [23]. The presence of contaminants such as chlorine, sulfur, and nitrogen in real waste can lead to complex reaction pathways and rapid catalyst poisoning, phenomena that are often underestimated in studies using simplified surrogates [24,25,26,27]. Therefore, validating the PCSR process with real-world feedstock is a prerequisite for industrial upscaling and the implementation of circular economy models [28].

This study addresses this gap by investigating the catalytic steam reforming of pyrolysis vapors derived from real MSW collected from 32 different municipalities. This extensive sampling provides a robust representation of the waste stream variability encountered in regional waste management systems. The novelty of this work lies in the scale and authenticity of the feedstock, providing critical insights for the future deployment of waste-to-hydrogen plants.

This work contributes to the advancement of MSW management and supports the strategic goals of the PNH2 by demonstrating a viable pathway for transforming environmental liabilities into high-value energy assets. In terms of originality, to the best of our knowledge, this study represents one of the first studies in Brazilian literature of the application of PSR and PCSR processes using real MSW from a municipal collection system, representing a significant advance in the development of sustainable technological routes for waste management and renewable hydrogen production.

## 2. Results and Discussion

### 2.1. MSW Characterization

**Figure 1:**
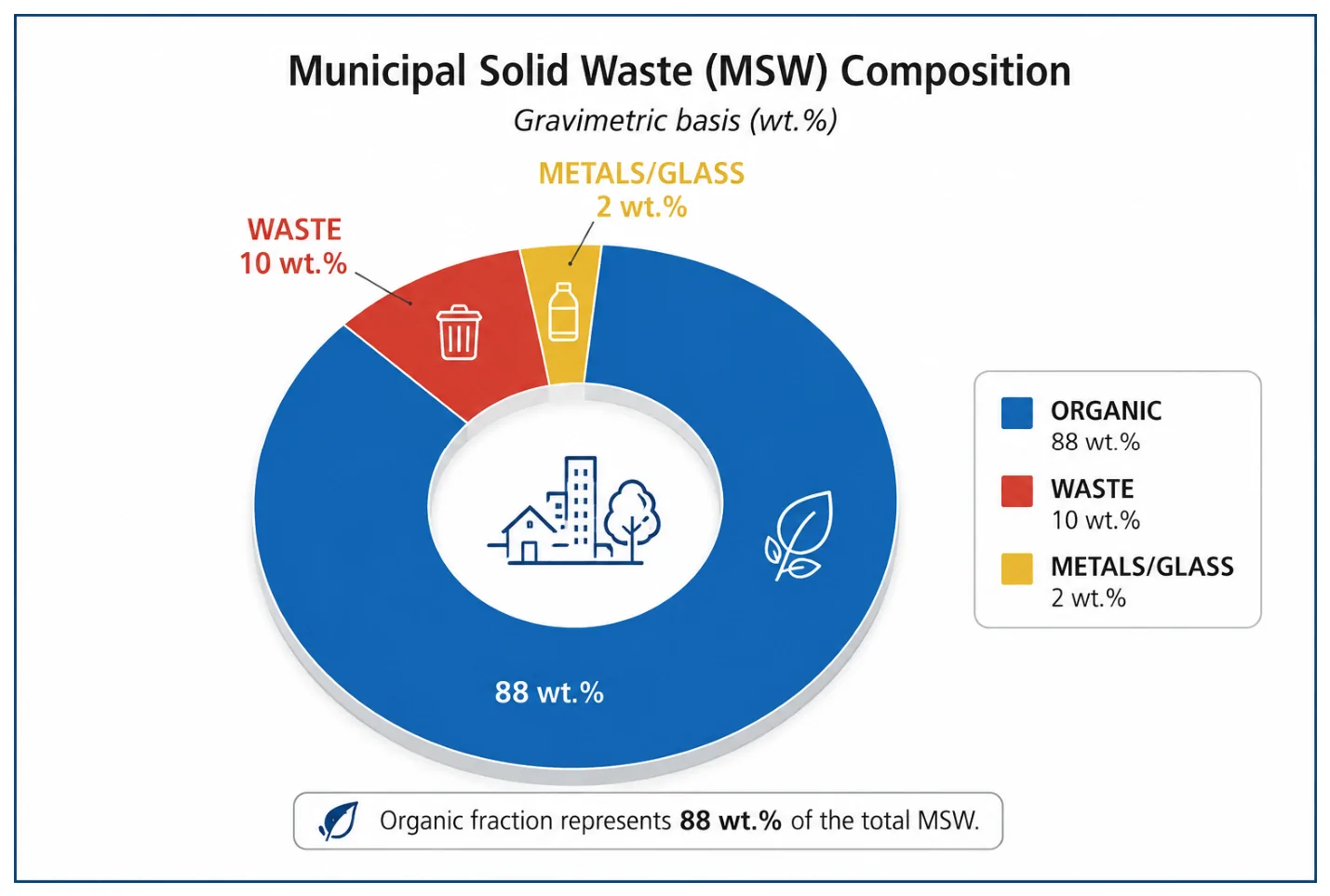
MSW gravimetric composition.

The gravimetric composition of the municipal solid waste (MSW) used in this study revealed a predominant organic fraction, as can be observed in Figure 1. The organic fraction (sample A), the main constituent fraction of the sample, is composed of food waste, pruning residues, bathroom waste, and disposable diapers. The waste fraction (sample B), referred to as reject material, is composed primarily of paper and polymeric materials that were incorrectly sent for collection. This high organic content suggests a significant potential for volatile matter release during the initial thermal degradation stage.

The MSW proximate and ultimate analyses are presented in Table 1. The samples presented volatile matter contents above 75 wt.%, favoring the use of thermochemical processes for their final disposal. The moisture contents obtained are close to literature data, being slightly higher than those from North American and European countries due to cultural factors and consumption patterns [29]. Waste with lower moisture contents promotes higher oil yields [30]. Moisture reduces oil viscosity, improving some characteristics; however, it decreases its calorific value [31]. The moisture content plays a fundamental role in the performance of the pyrolysis process, as it is a limiting factor due to the energy consumption required for its volatilization [32].

The carbon content of sample B is higher than that of sample A, due to the presence of polymeric material in its composition. Studies report a variation of 31.2–62.5 wt.% in carbon concentration in MSW, and the results of this work fall within this range [33,34,35,36]. The calorific value found in the MSW samples lies within the range of 12–27.6 MJ·kgMSW^−1^, as reported in the literature [17,35,37]. Low sulfur contents were observed in the samples (<0.16 wt.%), while higher chlorine values were found (>1 wt.%). Studies report chlorine contents in MSW ranging from 0.05 to 2.83 wt.%, reaching higher levels in the presence of PVC [24,25,26,27]. Due to the heterogeneous nature of MSW, the variability of chlorine content is influenced by the type of waste components [38].

**Figure 2:**
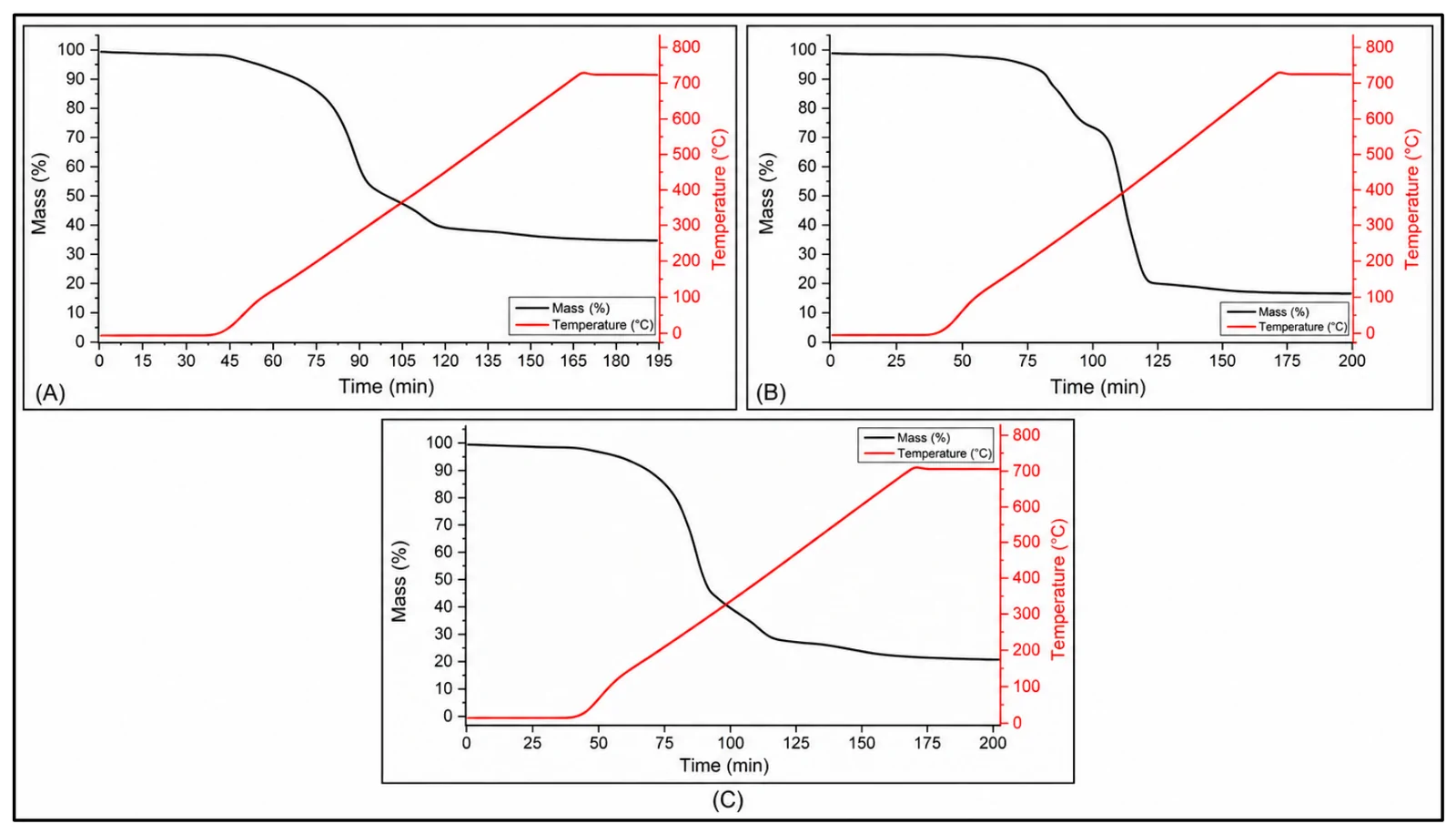
MSW thermogravimetric analysis: (**A**) Fraction A. (**B**) Fraction B. (**C**) Fraction C.

To determine the temperatures to be employed in the MSW pyrolysis experiments, thermogravimetric analysis of the samples was conducted. From the triplicate mean values, the curves presented in Figure 2 were obtained. The thermogravimetric curves indicate that the optimal temperature range lies below 500 °C, as this region exhibits the highest mass loss across all samples. Above this temperature, mass loss becomes negligible, signaling the complete release of volatile matter from the samples. At 500 °C, the mass losses for samples A, B, and C were 60.1%, 81.4%, and 69.9%, respectively. The lower mass loss observed for sample A is attributed to its higher ash content and lower volatile matter content.

The selection of 500 °C as the pyrolysis temperature is supported by the thermogravimetric analysis, which indicates that the volatile matter of the three MSW fractions is essentially fully released below this temperature (mass losses of 60.1%, 81.4% and 69.9% for fractions A, B and C at 500 °C, respectively). This behavior is consistent with the literature, which reports that the thermal decomposition of MSW occurs predominantly in the 220–560 °C range, with the volatile matter largely released by ~500 °C [39]. Operating the pyrolysis stage at higher temperatures (e.g., 700 °C) would not substantially increase volatile release for these feedstocks but would increase the energy demand and promote secondary cracking and repolymerization reactions that may reduce the selectivity toward the desired volatile products.

Reference [40] conducted the thermal analysis of MSW under an inert atmosphere (N_2_) at a heating rate of 20 °C·min^−1^, with the MSW consisting of organic and recyclable materials. The authors reported a mass loss of approximately 50% at 600 °C, whereas the results obtained for sample C in the present work were higher (approximately 76%). This discrepancy may be attributed to the high ash content reported by the authors (30.79%), combined with the distinct compositional profiles of MSW across different geographical regions.

### 2.2. Pyrolysis Yields

Pyrolysis experiments were conducted at a final temperature of 500 °C to evaluate the distribution of char and non-condensable gas products. The char yields obtained in this study (fraction A: 29.84% + 0.55; fraction B: 22.58% + 4.00; fraction C: 27.68% + 1.50) are consistent with values reported in the literature for the pyrolysis of municipal solid waste (MSW). Ref. [41] reported char yields ranging from 28.6 to 34.6 wt.% for MSW pyrolyzed at 400 °C. Similarly, ref. [42] obtained a char yield of 31 wt.% from household MSW subjected to pyrolysis at 450 °C, while [43] reported a char yield of 34.83 wt.% for laboratory-prepared MSW, formulated to simulate the physical composition of domestic waste (food waste, paper, and plastic materials), pyrolyzed at 550 °C. At higher temperatures, ref. [44] reported char yields between 32.4 and 38.7 wt.% for MSW pyrolyzed at 750 °C. In contrast, ref. [45] obtained lower char yields, ranging from 11.7 to 18.0 wt.%, for MSW composed predominantly of post-consumer plastic and textile wastes pyrolyzed at 550 °C. This behavior is consistent with the low solid residue formation typically observed during the pyrolysis of polymeric materials, for which char yields below 10 wt.% have been reported at 500 °C [46].

Beyond the quantitative yields, the solid co-product deserves consideration with respect to its expected composition and potential applications, which are directly relevant to the carbon balance, economic value and scalability of the proposed process. Based on the literature on MSW-derived biochars [47], the char produced at 500 °C is expected to consist predominantly of fixed carbon and inorganic constituents (ash), retaining a significant fraction of the feedstock carbon and the mineral matter (including alkali and alkaline-earth elements, as well as the chlorine and sulfur originally present in the waste). Owing to its carbonaceous structure and surface functionality, MSW-derived biochar has been proposed for a range of applications, including soil amendment and carbon sequestration, pollutant adsorption in water and soil remediation, and use as a solid fuel or process fuel in thermal integration schemes [48,49]. In the context of the present work, the biochar represents a valuable co-product of the PCSR route: its combustion (or gasification) can supply a substantial part of the thermal demand of the endothermic reforming stage, improving the overall energy balance, while its carbon content—when the char is used as a soil amendment instead of being combusted—contributes to carbon sequestration, further enhancing the carbon footprint of the process. The relatively high ash content of the MSW-derived char (consistent with the ash contents reported in Table 1) should, however, be taken into account when evaluating its suitability for specific applications, as it may limit its value in soil applications and increase the slagging risk in combustion systems. A dedicated characterization of the biochar—including elemental and proximate analysis, surface area, pH, FTIR and calorific value—was beyond the scope of this present study and is identified as a priority for future work, together with the assessment of its market value and end-use destinations in the framework of a full techno-economic and life-cycle assessment.

**Figure 3:**
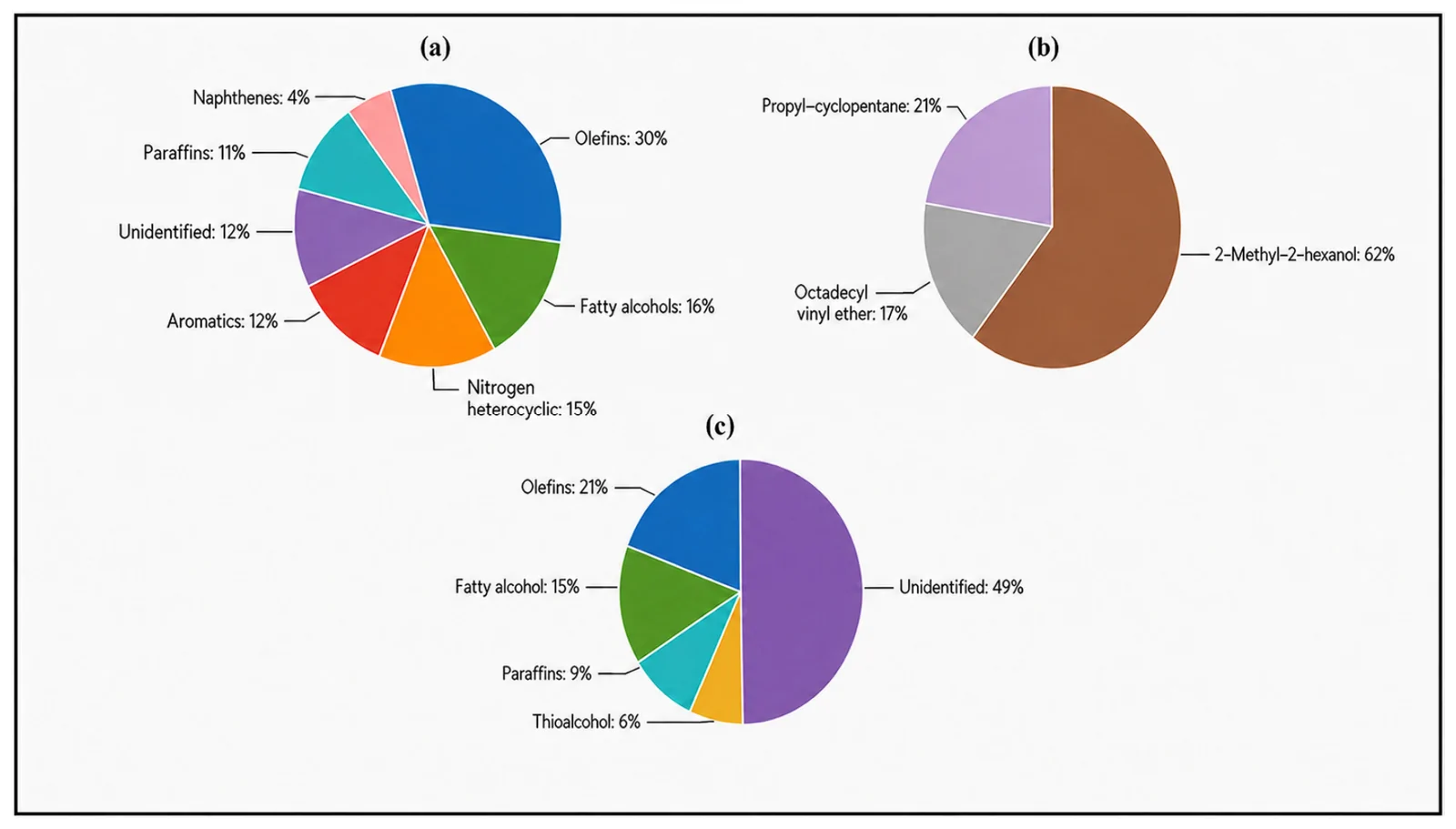
Chemical class distribution of condensable vapors (heavy phase) from MSW pyrolysis obtained by GC–MS: (**a**) Fraction A. (**b**) Fraction B. (**c**) Fraction C.

The identification of the components present in the heavy phase of the condensable vapors is presented in Figure 3. The analysis identified the presence of long-chain compounds (C7–C44), including paraffins, olefins, naphthenes, and aromatic compounds, among other groups. Such compounds were also identified by [17], who reported the presence of a wide range of components with C7–C34 chains in the heavy phase of the bio-oil produced from MSW pyrolysis at 500 °C. Studies report that the condensable vapors obtained from MSW pyrolysis contain polycyclic aromatic hydrocarbons (PAHs) and monoaromatic hydrocarbons (MAHs), alkanes and alkenes, as well as the presence of functional groups such as methyl, methoxyl, ethyl, and vinyl, produced during the fragmentation and formation of the benzene ring [42,50,51,52].

**Figure 4:**
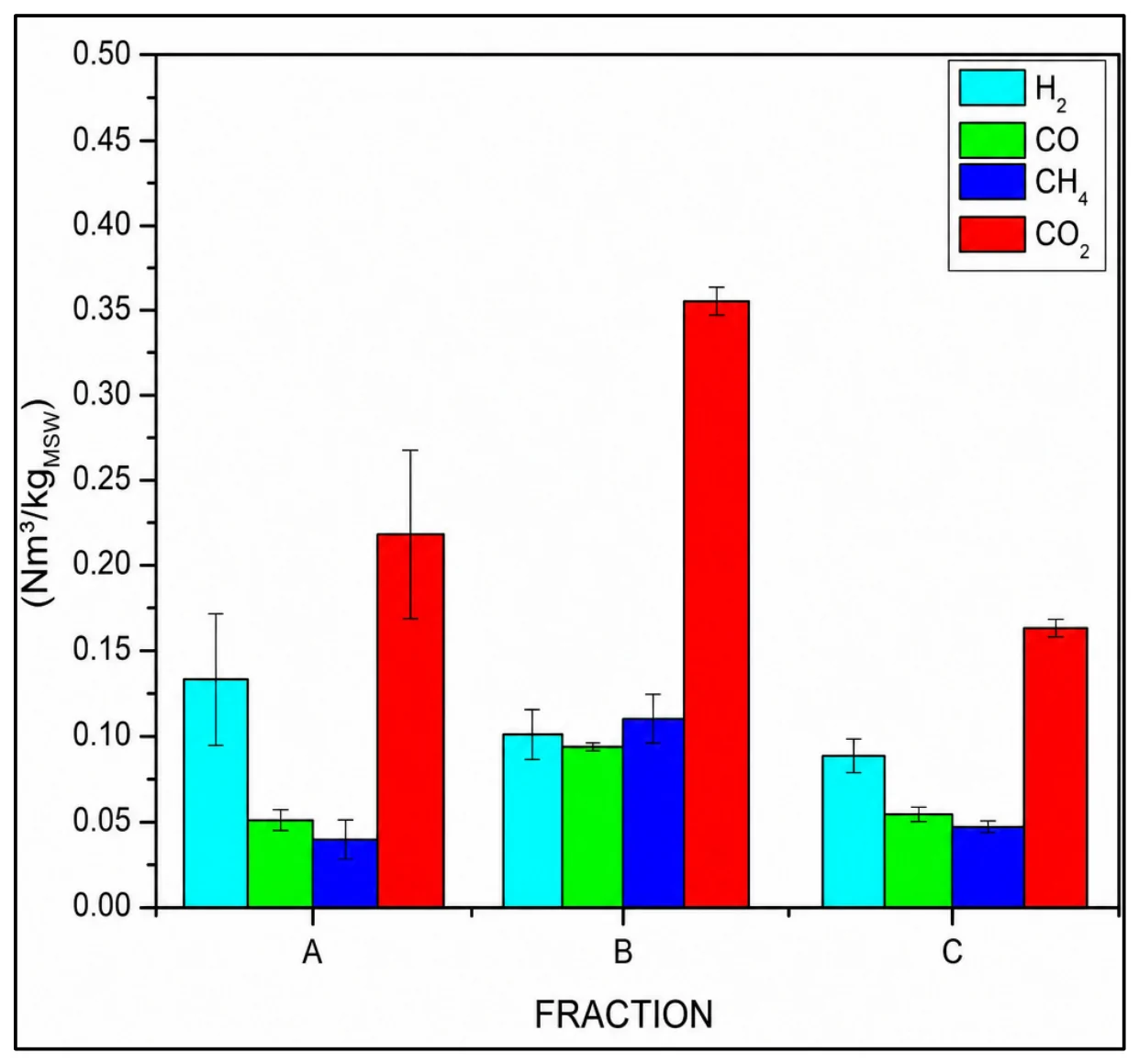
Non-condensable gas composition produced in the pyrolysis process. Organic-rich (A), polymeric-rich (B), and a mixed real collection fraction (C).

The components of the non-condensable gas produced in the MSW pyrolysis experiments were also evaluated, as presented in Figure 4. The non-condensable gas yield was determined on an N_2_-free basis and expressed as Nm^3^·kg^−1^. Fraction B exhibited the highest gas yield, reaching 0.66 ± 0.23 Nm^3^·kg^−1^, while producing the lowest char yield (22.58 ± 4.00 wt.%). In contrast, samples A and C generated lower gas yields of 0.44 ± 0.03 and 0.35 ± 0.16 Nm^3^·kg^−1^, respectively, accompanied by higher char yields of 29.84 ± 0.55 wt.% and 27.68 ± 1.50 wt.%, respectively. The superior gas production observed for sample B can be attributed to its higher volatile matter content and lower ash content, which favored the conversion of the organic fraction into gaseous products while reducing the amount of solid char.

The composition of the non-condensable gas produced during pyrolysis varied significantly among the fractions. Fraction B (polymeric) presented a higher content of light hydrocarbons, while Fraction A (organic) produced a greater content of CO and CO_2_, reflecting the distinct elemental composition of each fraction. Ref. [17] studied the pyrolysis of MSW at 500 °C collected from a landfill site and reported yields of approximately 0.05 Nm^3^H_2_·kgMSW^−1^, 0.08 Nm^3^CO·kgMSW^−1^, 0.02 Nm^3^CH_4_·kgMSW^−1^, and 0.06 Nm^3^CO_2_·kgMSW^−1^. The variation between the results reported by the authors and those obtained in the present work, depicted graphically in Figure 4, may be related to the MSW collection source. MSW retrieved from landfill cells has already undergone initial degradation, thus differing in composition. Ref. [51] reported that MSW pyrolysis at 500 °C yields a non-condensable gas in which CO_2_ is the major component, followed by CO, CH_4_, and H_2_. Ref. [53] evaluated the pyrolysis of healthcare waste (HCW) at 800 °C. H_2_ yields were reported to be in the range of 7.57–12.28 mmol H_2_·g HCW^−1^, corresponding to 0.17–0.27 Nm^3^ H_2_·kg HCW^−1^.

### 2.3. Pyrolysis and Steam Reforming (PSR)

**Figure 5:**
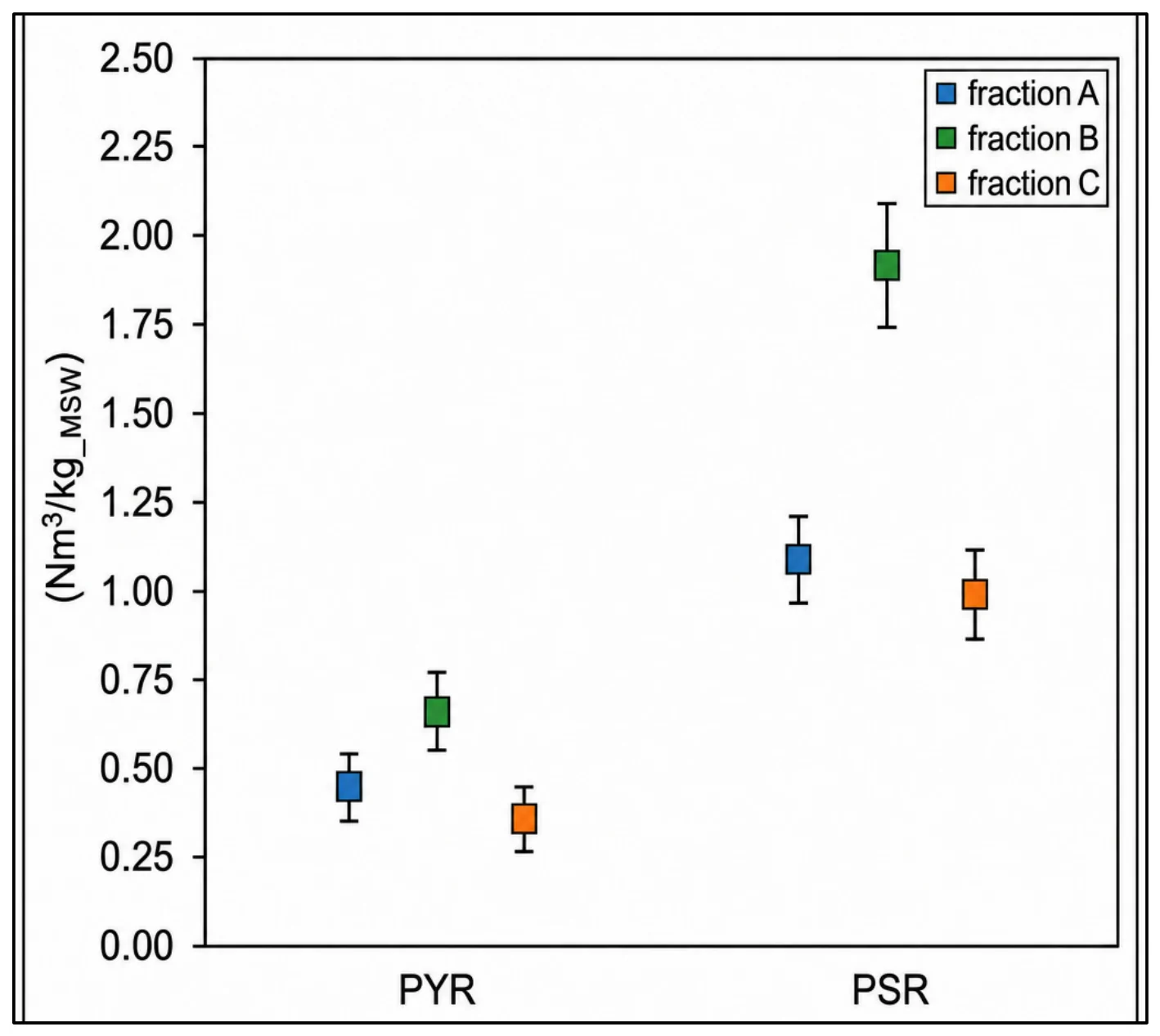
Non-condensable gas yields (Ygas) from pyrolysis (PYR) vs pyrolysis and steam reforming (PSR).

Coupling steam reforming in series with pyrolysis (PSR) promoted a significant increase in gas yield. Figure 5 presents the non-condensable gas yields (Ygas) from the pyrolysis (PYR) processes and the gas yields from the pyrolysis and steam reforming (PSR) in series for the three samples investigated in this study.

Regarding the pyrolysis process, the yields obtained in this work are also higher than those reported by the authors in [43], who, when evaluating the MSW pyrolysis process at different temperatures, reported a gas yield of 0.125 Nm^3^·kgMSW^−1^ at 500 °C. Ref. [17] studied the pyrolysis of MSW collected from a landfill at 500 °C and reported yields of approximately 0.05 Nm^3^H_2_·kgMSW^−1^, 0.08 Nm^3^CO·kgMSW^−1^, 0.02 Nm^3^CH_4_·kgMSW^−1^, and 0.06 Nm^3^CO_2_·kgMSW^−1^. The variation between the results reported by the authors and those found in this work may be related to the MSW collection source. MSW collected from landfill cells has already begun the degradation process, thus differing in composition.

The results obtained in the PSR process indicated an increase in gas yield compared to the pyrolysis process, with this increment being more pronounced for sample B, which is rich in polymeric materials. The results obtained were higher than those reported by [17]. The authors reported a gas yield of 0.31 Nm^3^·kg^−1^ from the PSR process (pyrolysis at 500 °C and steam reforming at 500 °C) of MSW. When the reforming temperature was increased to 550 °C and 600 °C, while maintaining the pyrolysis temperature at 500 °C, a gas yield of 0.40 Nm^3^·kg^−1^ was reported for both temperatures. It is suggested that the increase in gas yield is associated with the rise in steam reforming temperature, as the cracking of condensable vapor molecules is favored at high temperatures [20,54].

Table 2 presents the volumetric samples of the gas components produced in the PSR process. The gas yield increased from 0.35 Nm^3^·kgMSW^−1^ (pyrolysis) to 0.97 Nm^3^·kgMSW^−1^ in the PSR process for fraction C, representing an increase of approximately 177%. This increase is attributed to the conversion of condensable vapors and residual char into permanent gases via steam reforming reactions.

Fraction B (polymeric fraction) stood out with the highest volumetric H_2_ content, reaching 59.31%, and the highest H_2_ yield of up to 1.12 Nm^3^H_2_·kgMSW^−1^. This result is consistent with the higher elemental hydrogen content in polymers and the greater reactivity of long-chain hydrocarbons in steam reforming. Non-condensable gas yields are reported in Table 2. Ref. [51] reported that MSW pyrolysis at 500 °C produces a non-condensable gas with CO_2_ as the major component, followed by CO, CH_4_, and H_2_. Ref. [53] evaluated the pyrolysis of Healthcare Waste (HCW) at 800 °C. H_2_ yields were reported to be between 7.57 and 12.28 mmolH_2_·gHCW^−1^, i.e., 0.17–0.27 Nm^3^H_2_·kgHCW^−1^.

**Figure 6:**
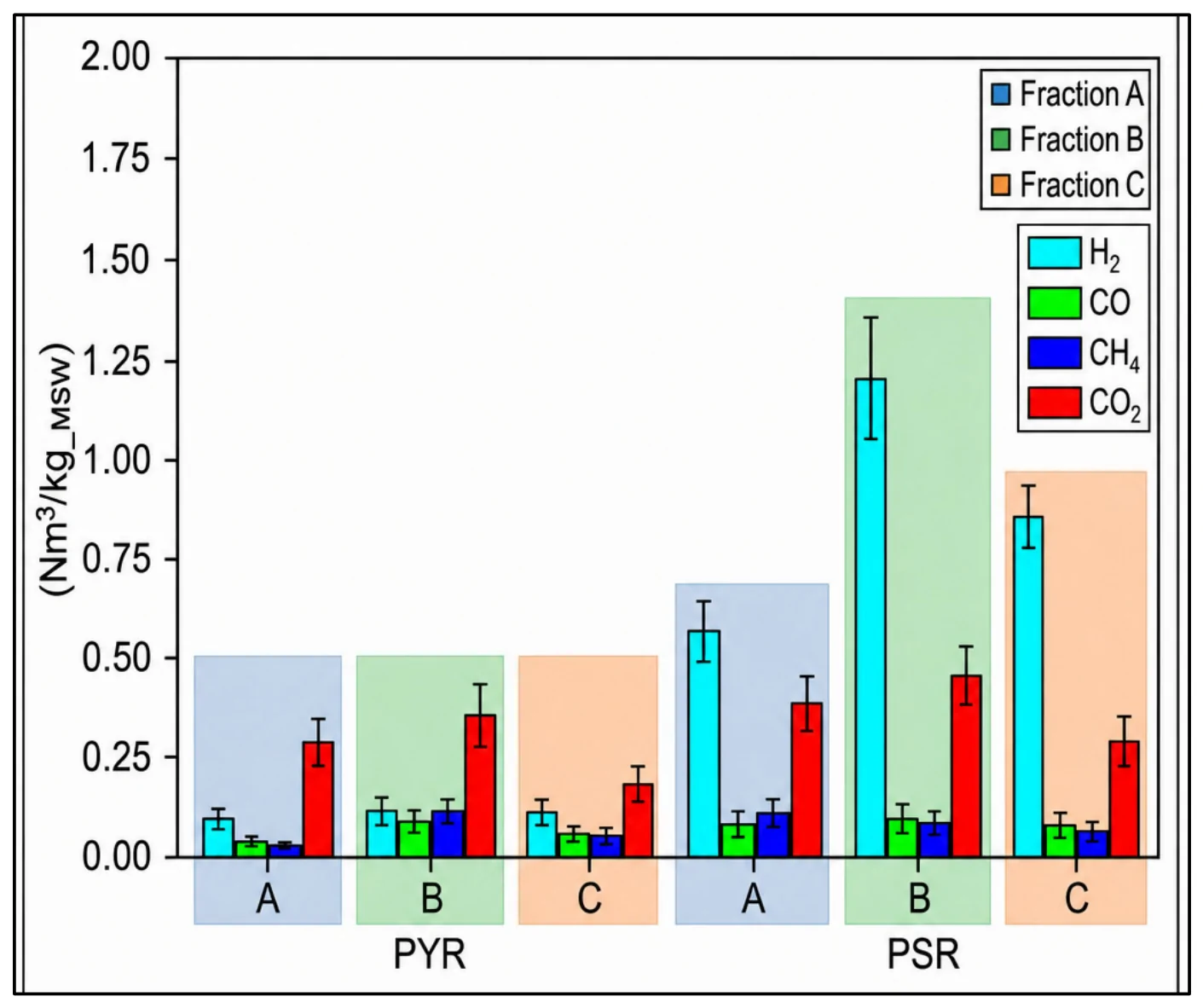
Non-condensable gas composition from pyrolysis (PYR) vs pyrolysis and steam reforming (PSR).

Figure 6 presents the non-condensable gas component yields from the PYR processes and the gas component yields from the PSR process for the three fractions investigated in this study. While in the PYR process the highest yield is observed for carbon dioxide across all investigated samples, in the PSR process the highest yield is hydrogen for all investigated samples. It is also noteworthy that carbon dioxide is the second component with the highest yield in the PSR process for all three fractions investigated in this study.

Ref. [42] studied the pyrolysis of MSW at 450 °C with thermal reforming (without catalyst) at 700 °C in series, reporting volumetric fractions of 36%, 12%, 14%, and 21% for H_2_, CO, CH_4_, and CO_2_, respectively. Ref. [17] reported a hydrogen volumetric fraction below 30% downstream of MSW pyrolysis (500 °C) and reforming (500–600 °C). When the data reported by the authors are compared to the results of this study, it is verified that the use of steam reforming increased the H_2_ yield, producing a hydrogen-rich gas.

Results obtained in this study suggest a volumetric fraction exceeding 50% of H_2_ from MSW. Ref. [17] reported H_2_ volumetric fractions between 32% and 42% from the MSW pyrolysis (500 °C) and reforming (500–600 °C) process, which are lower than those obtained in this study (52–59%). Ref. [55] studied steam gasification at 800 °C in the absence of a catalyst of a polymeric fraction contained in recyclable material rejects, reporting a hydrogen volumetric fraction of approximately 73%. That study demonstrates that the presence of polymeric materials plays an important role in the production of a hydrogen-rich gas, which can also be observed in the present study, where sample B, containing predominantly polymeric material residues, presented the highest hydrogen yield.

### 2.4. Pyrolysis and Catalytic Steam Reforming (PCSR)

**Figure 7:**
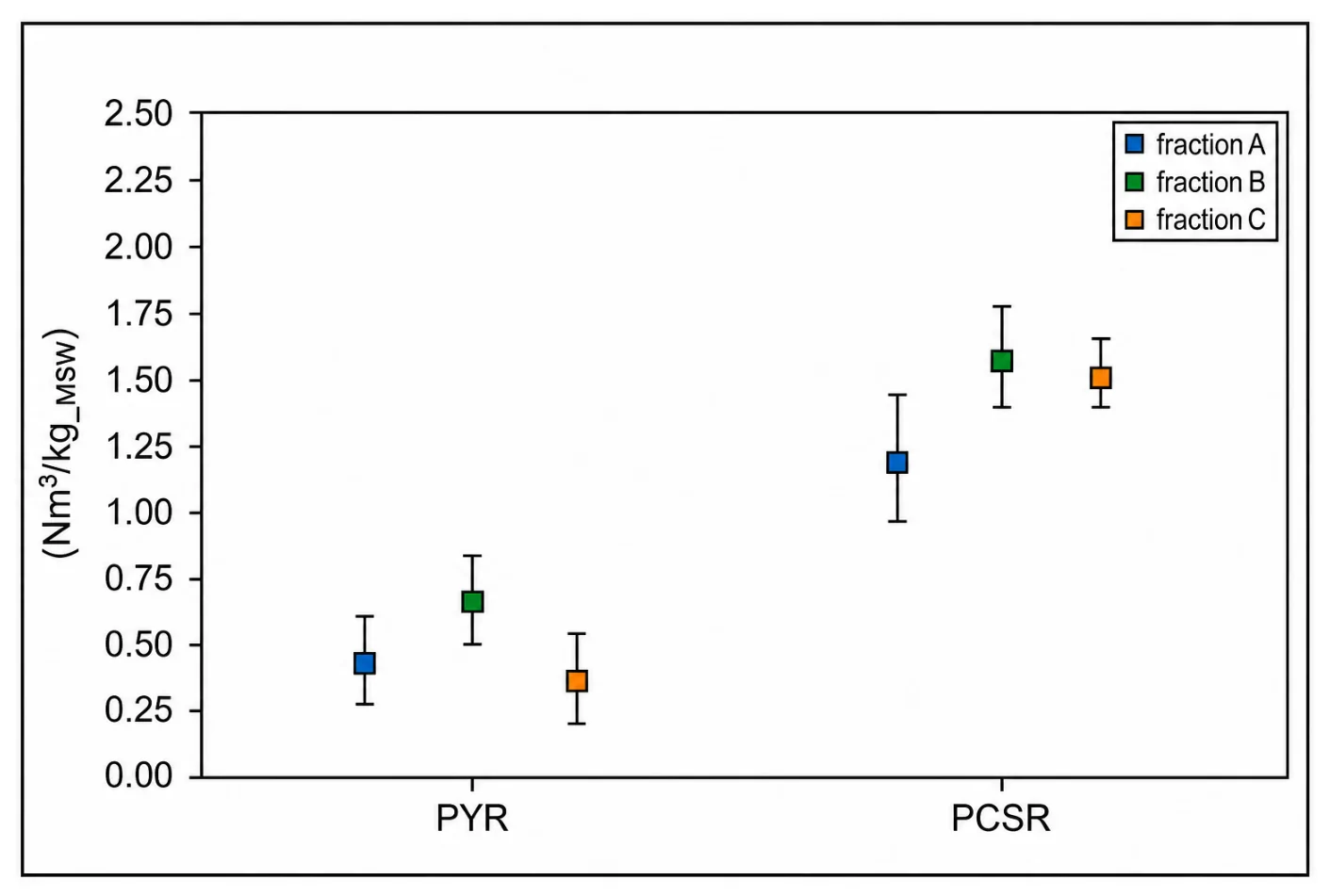
Non-condensable gas yields (Ygas) from pyrolysis (PYR) vs pyrolysis and catalytic steam reforming (PCSR).

Figure 7 presents the non-condensable gas yields from the pyrolysis (PYR) processes and the gas yields from the pyrolysis and catalytic steam reforming (PCSR) processes for the three fractions investigated in this study. The introduction of the commercial Ni/Al_2_O_3_ catalyst in the reforming bed at 900 °C (PCSR) promoted the highest gas yield among all processes studied. According to Table 2, gas yield reached 1.50 Nm^3^·kgMSW^−1^ for fraction C, exceeding the value obtained in PSR (0.97 Nm^3^·kgMSW^−1^) by 54% and the pyrolysis value (0.35 Nm^3^·kgMSW^−1^) by 329%. This increase demonstrates the efficiency of the Ni/Al_2_O_3_ catalyst in promoting additional conversion of residual hydrocarbons and char.

To isolate the intrinsic catalytic contribution of the Ni/Al_2_O_3_ catalyst, the PSR process (pyrolysis at 500 °C followed by steam reforming at 900 °C in the absence of catalyst) was employed as the thermal baseline, since it was conducted under conditions identical to the PCSR process—same pyrolysis stage, same reforming temperature and same steam flow rate—differing only in the presence of the catalyst. The direct comparison between PSR and PCSR therefore isolates the catalytic effect at the same operating temperature. The catalytic contribution is evidenced by the higher H_2_ yields (from 0.60 to 1.10, from 1.10 to 1.40 and from 0.85 to 1.30 Nm^3^·kg^−1^ for fractions A, B and C, respectively), the near-complete elimination of CO (from 5.5 to 6.0% vol. in PSR to 0.5% vol. in PCSR for all fractions) and the increase in H_2_ + CO_2_ purity (from ~80% vol. in PSR to 96.1–98.0% vol. in PCSR), reflecting the catalytic promotion of the water–gas shift and methane reforming reactions over the Ni active sites. It should be noted, however, that in the PSR configuration the reforming reactor was operated without a packed bed, whereas the PCSR reactor contained the catalytic bed, so that the bed hydrodynamics differ between the two configurations. A control experiment employing an inert packed bed (e.g., bare SiC or quartz spheres) at 900 °C would further strengthen this comparison by replicating the packed-bed hydrodynamics in the absence of catalytic activity.

Ref. [17] reported hydrogen yields between 0.36 and 0.46 Nm^3^·kg^−1^ for the pyrolysis process at 500 °C, followed by catalytic steam reforming in the temperature range of 500–600 °C, using char as a catalyst. Ref. [53] investigated H_2_ production via PCSR at 800 °C from healthcare waste (HCW), using CaO as a catalyst. The hydrogen yield increased by approximately 52%, while the pyrolysis oil yield was reduced by approximately 30%. The authors reported a hydrogen yield of 12.28 mmol H_2_·g HCW^−1^, corresponding to 0.27 Nm^3^·kg HCW^−1^.

In addition to carbon deposition and Ni sintering, the chlorine content of the feedstock constitutes a critical deactivation pathway for the Ni/Al_2_O_3_ catalyst. The MSW fractions employed in this study contain significant chlorine contents (1.28, 1.18 and 1.22 wt.% for fractions A, B and C, respectively), within the range reported in the literature for real MSW (0.05–2.83 wt.%) [24,25,26,27]. During the pyrolysis stage, chlorine is released predominantly as HCl, which is carried with the volatile stream into the reforming reactor at 900 °C. HCl is a potent poison for Ni-based catalysts, acting through two main mechanisms: the formation of volatile nickel chlorides (NiCl_2_), which promotes the loss of the active metal from the catalyst surface, and the competitive adsorption of chlorine species on the Ni active sites, which reduces the number of sites available for steam reforming and water–gas shift reactions. Despite the significant chlorine content of the feedstock, the catalyst maintained sufficient activity throughout each individual run, as reflected by the consistently high gas and H_2_ yields achieved across the three fractions (Table 2). Nevertheless, the cumulative effect of chlorine poisoning on the long-term stability of the catalyst cannot be assessed from the present single-run experiments. The verification of the post-reaction Ni sites—including XPS to detect chlorine species and the oxidation state of Ni, XRD to identify nickel chloride phases and Ni sintering, and SEM/TEM/EDS to assess morphological changes and chlorine distribution—is therefore identified as a priority for future work, together with the evaluation of the chlorine tolerance and regeneration strategies of the catalyst for the industrial implementation of the PCSR processing of real municipal solid waste.

**Figure 8:**
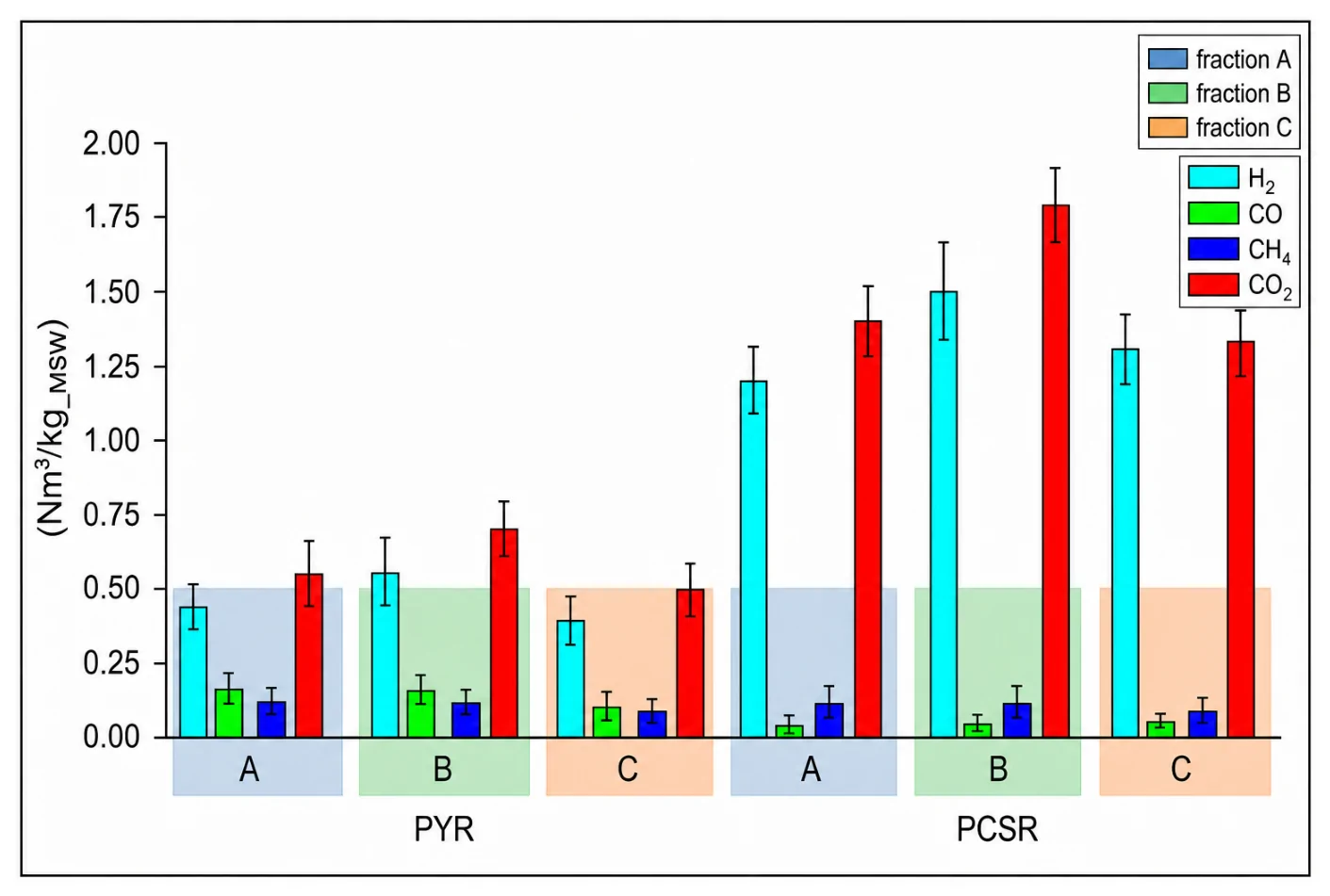
Non-condensable gas composition from pyrolysis (PYR) vs pyrolysis and catalytic steam reforming (PCSR).

Figure 8 presents the non-condensable gas component yields from the pyrolysis (PYR) processes and the gas component yields from the pyrolysis and catalytic steam reforming (PCSR) processes for the three samples investigated in this study. The gas generated in the PCSR process is composed primarily of H_2_ and CO_2_, with negligible yields of CO and CH_4_.

A quantitative comparison of the gas composition before and after catalytic reforming substantiates the suitability of the PCSR gas for hydrogen purification. Table 2 presents the complete gas composition data (H_2_, CO, CO_2_, CH_4_) for the PYR, PSR and PCSR stages, together with the gas yield and H_2_ yield. Three quantitative indicators demonstrate the improvement promoted by the Ni/Al_2_O_3_ catalyst. First, the CO content decreased from 11.6, 14.2 and 15.5% vol. in the PYR stage to 0.5% vol. for all fractions in the PCSR stage, corresponding to reductions of 95.7%, 96.5% and 96.8% for fractions A, B and C, respectively, relative to the PYR stage. This near-complete elimination of CO is attributed to the promotion of the water–gas shift reaction over the Ni active sites. Second, the CH_4_ content decreased from 9.1, 16.7 and 13.4% vol. in the PYR stage to 1.9, 1.5 and 3.4% vol. in the PCSR stage, corresponding to reductions of 79.1%, 91.0% and 74.6%, respectively, consistent with the promotion of methane steam reforming and CH_4_ decomposition. Third, the combined H_2_ + CO_2_ fraction—which represents the gas that can be directly processed for H_2_ recovery after CO_2_ removal—increased from 79.3%, 69.0% and 71.1% vol. in the PYR stage to 97.6%, 98.0% and 96.1% vol. in the PCSR stage for fractions A, B and C, respectively. The PCSR gas is therefore almost entirely composed of H_2_ and CO_2_, with only ~2–4% vol. of residual CO and CH_4_, which greatly facilitates downstream H_2_ separation and purification. In parallel, the H_2_ yield increased from 0.60, 0.60 and 0.35 Nm^3^·kg^−1^ (PYR) to 1.10, 1.40 and 1.30 Nm^3^·kg^−1^ (PCSR) for fractions A, B and C, respectively, confirming that the catalytic stage simultaneously enhances H_2_ production and gas purity.

The H_2_ yield maximum was 0.60 Nm^3^H_2_·kgMSW^−1^ in PYR and 1.54 Nm^3^H_2_·kgMSW^−1^ in PCSR. The gas produced exhibits higher purity in terms of H_2_ + CO_2_, with very low levels of contaminants (CO and CH_4_), which facilitates subsequent H_2_ separation and purification steps. The high concentration of H_2_ and CO_2_ at the expense of CO concentration derives from the occurrence of the water–gas shift reaction (CO + H_2_O ↔ H_2_ + CO_2_), a phenomenon that has been reported in the literature [51]. Furthermore, a significant reduction in CO and CH_4_ concentrations is observed in the steam reforming stage, suggesting that methane steam reforming and water–gas shift reactions are favored.

The mechanistic interpretation of the catalytic effect of Ni/Al_2_O_3_ is supported by the analysis of the gas evolution trends across the PYR → PSR → PCSR sequence, which provide indirect evidence of the reaction pathways promoted over the Ni active sites. Three main contributions can be identified. First, the pronounced reduction in CH_4_ content from the PSR to the PCSR stage (e.g., from 8.5 to 1.9% vol. for fraction A, from 7.5 to 1.5% vol. for fraction B and from 8.7 to 3.4% vol. for fraction C), combined with the simultaneous increase in H_2_ yield, is consistent with the promotion of methane steam reforming (CH_4_ + H_2_O → CO + 3H_2_) and, to a lesser extent, of CH_4_ decomposition (CH_4_ → C + 2H_2_) over the nickel surface. Second, the substantial increase in total gas yield from the PSR to the PCSR stage (e.g., from 0.97 to 1.50 Nm^3^·kg^−1^ for fraction C) indicates that the Ni catalyst promoted the cracking and steam reforming of heavy hydrocarbons, oxygenated compounds and tars that were not fully converted by thermal reforming alone, releasing additional permanent gases. Third, the CO content decreased to negligible levels in the PCSR stage (0.5% vol. for all fractions) while CO_2_ increased markedly (e.g., from 27.8 to 56.2% vol. for fraction C), which is the characteristic signature of the water–gas shift reaction (CO + H_2_O ↔ CO_2_ + H_2_) promoted over the Ni active sites at 900 °C under excess steam. The combination of these pathways explains the high H_2_ + CO_2_ purity of the PCSR gas and the negligible CO and CH_4_ levels observed in Figure 8. It should be noted, however, that the present study did not include operando characterization of the active Ni species or of reaction intermediates; such analyses (e.g., in situ XRD, XPS or DRIFTS) are identified as necessary to provide direct evidence of the active phase and to further validate the proposed mechanism.

The magnitude of the catalytic enhancement promoted by the Ni/Al_2_O_3_ catalyst was found to be strongly dependent on the feedstock composition. For fraction A (organic-rich), the H_2_ yield increased from 0.60 Nm^3^·kg^−1^ (PSR) to 1.10 Nm^3^·kg^−1^ (PCSR), corresponding to an increment of approximately 83%, whereas for fraction B (polymeric) the increase was only from 1.10 to 1.40 Nm^3^·kg^−1^ (+27%). This behavior reflects the distinct elemental and chemical nature of each fraction. Fraction B, composed predominantly of polyolefins, exhibits a high H/C ratio and a low oxygen content, and its long-chain hydrocarbons are readily cracked under thermal steam reforming, generating H_2_ efficiently even in the absence of a catalyst; the Ni catalyst therefore provides a comparatively smaller incremental gain, acting mainly by promoting the water–gas shift of the CO produced. In contrast, fraction A, rich in cellulose, hemicellulose, lignin and food waste, generates a higher proportion of oxygenated compounds and refractory tars, which are less efficiently converted by thermal reforming alone. For this fraction, the Ni active sites play a decisive role by promoting the cracking of oxygenates and tars, methane steam reforming and the water–gas shift, resulting in a substantially larger incremental H_2_ yield. These results demonstrate that the catalytic route is most impactful precisely where thermal steam reforming is least effective—i.e., for oxygen-rich, tar-laden feedstocks—and highlight the importance of feedstock composition in the design of waste-to-hydrogen processes.

The performance of the commercial Ni/Al_2_O_3_ catalyst in the PCSR process is consistent with the behavior reported in the literature for Ni-based catalysts applied to the reforming of MSW and plastic waste. Irfan et al. [52] reported hydrogen concentrations between 45 and 50% in the gas obtained from MSW pyrolysis at 850 °C followed by steam reforming over nickel catalysts, values comparable to those achieved in the present work (32–39% vol. in the PCSR stage). Similarly, recent reviews on the gasification and reforming of MSW confirm that, among transition-metal catalysts, nickel exhibits the highest activity for hydrogen production (Ni > Co > Fe), owing to its high capacity for C–C and C–H bond cleavage and for promoting the water–gas shift reaction. The use of Ni/Al_2_O_3_ in the present study therefore aligns with the most effective catalytic systems reported for waste-to-hydrogen routes, while the moderate surface area of the commercial catalyst (10.3 m^2^·g^−1^) indicates that the observed performance is governed primarily by the intrinsic activity of the Ni active phase rather than by the support surface area. This finding is relevant for process economics, as it demonstrates that high hydrogen yields can be achieved with a commercially available, low-cost catalyst without the need for highly engineered supports.

The markedly different temperatures employed in the two stages (500 °C for pyrolysis and 900 °C for catalytic steam reforming) reflect the distinct thermodynamic requirements of each reaction. The pyrolysis stage was set at 500 °C because the thermogravimetric analysis demonstrated that the volatile matter of the MSW fractions is essentially completely released at this temperature, so that higher temperatures would only increase the energy demand without a corresponding gain in volatile yield. In contrast, the steam reforming stage was operated at 900 °C because methane steam reforming (CH_4_ + H_2_O → CO + 3H_2_) is strongly endothermic and its equilibrium conversion increases with temperature, and because higher temperatures favor the conversion of refractory tars and heavy hydrocarbons released from the heterogeneous real MSW [56]. Although a common operating temperature near 700 °C would simplify reactor design and heat integration, it would be suboptimal for both stages: it would not increase volatile release in the pyrolysis stage, and it would reduce the thermodynamic driving force for methane reforming and tar conversion in the reforming stage, potentially lowering the H_2_ yield and gas purity. The two-stage configuration with distinct temperatures therefore represents a deliberate trade-off between minimizing the energy demand of the pyrolysis stage and maximizing the syngas conversion and H_2_ yield in the reforming stage.

The marked reduction in CO and CH_4_ concentrations observed in the PCSR stage, together with the enrichment of the gas in H_2_ and CO_2_, can be rationalized in terms of the main reaction pathways promoted over the Ni active sites. In the catalytic steam reforming stage, the long-chain hydrocarbons and oxygenated compounds produced during pyrolysis undergo steam reforming, generating CO and H_2_ (C_n_H_m_ + nH_2_O → nCO + (n + m/2)H_2_). The CO thus formed is subsequently converted through the water–gas shift reaction (CO + H_2_O ↔ CO_2_ + H_2_), which is favored at the reforming temperature of 900 °C and over the Ni surface, leading to the simultaneous increase in H_2_ and CO_2_ and the depletion of CO. In parallel, methane produced during pyrolysis is converted via methane steam reforming (CH_4_ + H_2_O → CO + 3H_2_), which accounts for the pronounced decrease in CH_4_ content from the PSR to the PCSR stage (e.g., from 8.5 to 1.9% vol. for fraction A). The combination of these pathways explains why the PCSR gas is composed primarily of H_2_ and CO_2_, with negligible yields of CO and CH_4_, as observed in Figure 8. This mechanistic interpretation is consistent with the literature, which attributes the high H_2_ + CO_2_ purity of the gas to the promotion of the water–gas shift and methane reforming reactions over nickel active sites [47,52]. 

Reference [57] reported hydrogen concentrations between 45 and 50% in the gas obtained from MSW pyrolysis at 850 °C, followed by steam reforming with nickel catalysts. Ref. [55] studied steam gasification of the polymeric fraction contained in the reject of recyclable materials at 800 °C, using CaO as a catalyst, and also reported a hydrogen volume fraction of approximately 73%.

Table 3 compares the hydrogen yields and gas compositions obtained in the present study with those reported in representative recent waste-to-hydrogen investigations. The PCSR process achieved hydrogen yields of 1.10–1.40 Nm^3^·kg^−1^ from real, heterogeneous MSW, which are competitive with those reported for MSW-derived feedstocks over Ni-based or char-based catalysts, and are remarkable given that the feedstock was not a synthetic mixture or a single plastic type. The higher yields reported for plastic-rich feedstocks (2.28–3.38 Nm^3^·kg^−1^) reflect the intrinsically higher hydrogen content and lower oxygen content of polyolefins, which favor H_2_ production during reforming. Conversely, the H_2_ concentration in the present PCSR gas (32–39% vol.) is lower than the maximum values reported for optimized char-based systems (up to 71% vol.), which is attributed to the higher CO_2_ content arising from the water–gas shift reaction over the Ni catalyst and to the oxygen-rich, heterogeneous nature of real MSW. These results highlight the trade-off between absolute H_2_ yield and feedstock representativeness, and demonstrate that the PCSR route offers a robust, industrially relevant pathway for waste-to-hydrogen conversion.

Although the PCSR process demonstrated high and reproducible gas and hydrogen yields across the different MSW fractions, the stability of the Ni/Al_2_O_3_ catalyst under the severe reforming conditions employed (900 °C) deserves consideration. At this temperature, Ni-based catalysts are susceptible to deactivation by sintering of the metallic phase, oxidation/reduction cycling and carbon deposition, which progressively reduce the number of active sites and the reforming activity. In the present study, each experiment was carried out in batch mode over a single run, and the catalyst was not subjected to repeated reaction–regeneration cycles; therefore, a quantitative evaluation of deactivation was outside the scope of this work. It should be noted, however, that the catalyst maintained sufficient activity throughout each individual run, as reflected by the consistently high gas and H_2_ yields obtained, and that two features of the experimental setup contributed to mitigating carbon deposition: the alumina guard bed placed below the catalytic section, which protects the active phase from particulate matter and heavy tars, and the excess steam supply (steam flow rate of 0.5 kg·h^−1^), which favors the gasification of carbonaceous residues and the water–gas shift reaction [19]. A comprehensive characterization of the spent catalyst—including XRD, SEM/TEM, Raman spectroscopy, TGA and XPS—is therefore identified as a necessary next step to quantify Ni sintering, redox changes and coke deposition, and to establish the long-term stability and regeneration strategies required for the industrial implementation of the PCSR route.

A quantitative assessment of the tar conversion over the Ni/Al_2_O_3_ catalyst was not performed in the present study. The condensable phase was characterized semi-quantitatively by GC–MS (Figure 3), which identified long-chain compounds (C7–C44) including paraffins, olefins, naphthenes and aromatic species (monoaromatic and polycyclic aromatic hydrocarbons), in agreement with previous reports [17,41,46,47,48]. Although a direct mass balance of the heavy phase across the PYR, PSR and PCSR stages was not resolved, indirect evidence indicates extensive conversion of the condensable vapors during the catalytic stage: the total gas yield increased substantially from PYR to PCSR (from 0.44, 0.66 and 0.35 Nm^3^·kg^−1^ in PYR to 1.18, 1.54 and 1.50 Nm^3^·kg^−1^ in PCSR for fractions A, B and C, respectively), which is attributed to the steam reforming and cracking of condensable vapors and residual char into permanent gases over the Ni active sites; moreover, the PCSR gas reached H_2_ + CO_2_ purities of 96.1–98.0% vol. with CO reduced to 0.5% vol., consistent with the deep conversion of heavy hydrocarbons and tars. Nevertheless, the exact tar conversion efficiency requires dedicated quantification and is identified as a priority for future work.

It should be acknowledged that the present study did not include a thermodynamic equilibrium analysis of the catalytic steam reforming stage. Whereas the pyrolysis stage proceeds under kinetically controlled, non-equilibrium conditions, the catalytic steam reforming of the pyrolysis volatiles operates under conditions in which thermodynamic equilibrium exerts a dominant influence, particularly at the high reforming temperature of 900 °C. A quantitative comparison between the experimental gas compositions and the equilibrium distributions—obtained, for instance, by the Gibbs free energy minimization method—would allow the extent to which the reforming stage approaches the thermodynamic limit to be assessed, providing valuable information on process efficiency, catalyst performance and the remaining opportunities for optimization. Such an analysis requires the precise definition of the effective steam-to-carbon ratio and of the elemental balance of the volatile stream entering the reforming reactor, which are not fully resolved in the present batch-mode experiments. The thermodynamic equilibrium analysis is therefore identified as a priority for future work, together with the dedicated time-on-stream tests, the spent-catalyst characterization and the tar conversion quantification, to complete the experimental framework required for the industrial validation of the PCSR processing of real municipal solid waste.

It should be noted that the present study focused on the non-condensable gas products, which constitute the central objective of the PCSR route. The solid fraction (char) yields are reported in Section 2.2, and the condensable fraction was characterized semi-quantitatively by GC–MS (Figure 3), which identified long-chain compounds (C7–C44) including paraffins, olefins, naphthenes and aromatic species. However, a quantitative mass balance of the condensable (liquid) phase across the PYR, PSR and PCSR stages was not resolved in this study. The indirect evidence of extensive conversion of the condensable vapors during the catalytic stage—namely the substantial increase in total gas yield from PYR to PCSR (from 0.44, 0.66 and 0.35 Nm^3^·kg^−1^ in PYR to 1.18, 1.54 and 1.50 Nm^3^·kg^−1^ in PCSR for fractions A, B and C, respectively) and the H_2_ + CO_2_ purity of 96.1–98.0% vol. with CO reduced to 0.5% vol.—is consistent with the deep conversion of heavy hydrocarbons and tars over the Ni active sites. The gravimetric determination of the condensable (tar) phase and the completion of the overall mass balance are identified as priorities for future work.

With respect to the mass balance of the experiments, the non-condensable gas products were quantified in terms of volumetric yield and composition (Table 2), and the solid fraction (biochar) was quantified gravimetrically (Section 2.2). The condensable (liquid) phase, however, was characterized semi-quantitatively by GC–MS (Figure 3) but its gravimetric yield was not resolved, and the elemental composition of the biochar was not determined in this study. A complete closure of the overall mass balance—and of the carbon balance—across the gas, liquid and solid fractions therefore cannot be rigorously established with the presently available data. This limitation notwithstanding, the indirect evidence of extensive carbon conversion during the catalytic stage—namely the substantial increase in total gas yield from PYR to PCSR (from 0.44, 0.66 and 0.35 Nm^3^·kg^−1^ in PYR to 1.18, 1.54 and 1.50 Nm^3^·kg^−1^ in PCSR for fractions A, B and C, respectively) and the H_2_ + CO_2_ purity of 96.1–98.0% vol. with CO reduced to 0.5% vol. in the PCSR gas—is consistent with the deep conversion of heavy hydrocarbons and tars over the Ni active sites. The completion of the overall mass balance and of the carbon balance, including the gravimetric determination of the condensable (tar) phase and the elemental analysis of the biochar, is identified as a priority for future work.

Regarding the reporting of catalyst performance, the gas and H_2_ yields are expressed on a feedstock basis (Nm^3^·kgMSW^−1^) throughout the manuscript. Given the known mass of the commercial Ni/Al_2_O_3_ catalyst (9.8 wt.% Ni) employed in the PCSR experiments, these yields can be readily normalized to the catalyst mass or to the active Ni content, which would facilitate comparison with other studies and provide a more direct assessment of catalyst productivity. This normalization is a straightforward extension of the present data and is suggested for future comparative analyses.

### 2.5. Preliminary Energy and Carbon Assessment

A preliminary assessment of the energy and carbon performance of the PCSR process was carried out to evaluate whether the hydrogen production benefit can compensate for the additional energy demand of the high-temperature reforming stage. The chemical energy contained in the H_2_ produced in the PCSR stage (HHV H_2_ = 10.8 MJ·Nm^−3^) was estimated as 11.9, 15.1 and 14.0 MJ·kg^−1^ for fractions A, B and C, respectively. The estimated thermal demand of the process comprises the sensible heat required to heat the feedstock from ambient temperature to the pyrolysis temperature of 500 °C (~0.95 MJ·kg^−1^, assuming a specific heat of ~2.0 kJ·kg^−1^·K^−1^) and the latent heat required to vaporize the feedstock moisture (1.16, 0.15 and 1.08 MJ·kg^−1^ for fractions A, B and C, reflecting their moisture contents of 51.4%, 6.5% and 47.9%). On an industrially relevant basis with a steam-to-carbon ratio of ~1.5, the energy required for steam generation was estimated at ~2.4–5.0 MJ·kg^−1^ depending on the fraction. The ratio of H_2_ chemical energy to the estimated process thermal demand was approximately 2.5–3.0, indicating that the energy carried by the produced H_2_ can exceed the estimated thermal demand even before accounting for the energy value of the co-products (biochar and non-condensable gas). It should be noted, however, that this preliminary balance does not include reactor heat losses or the electrical energy required for heating in the bench-scale configuration, and that a rigorous energy audit is required for a definitive assessment.

From a carbon perspective, the PCSR route avoids both the emissions associated with the substituted grey hydrogen produced by steam methane reforming (SMR), which emits 10–12 kg CO_2_e per kg H_2_ [60], and the methane emissions avoided by diverting MSW from landfill (GWP100 ≈ 27–28) [61]. The avoided emissions were estimated at approximately 1.0–1.6 kg CO_2_e·kg^−1^ from H_2_ substitution and 0.5–1.0 kg CO_2_e·kg^−1^ from landfill diversion, totaling ~1.5–2.6 kg CO_2_e·kg^−1^, which exceeds the estimated process emissions of ~0.9–1.2 kg CO_2_e·kg^−1^ (assuming heat supplied by natural gas). The net carbon balance is therefore positive, and is even more favorable when renewable electricity is used, as in the Brazilian energy matrix. This is consistent with recent life-cycle assessment studies reporting that waste-to-hydrogen routes consistently exhibit lower greenhouse gas emissions than SMR, with carbon-negative performance achievable under specific assumptions (renewable electricity, avoided landfill emissions and biogenic carbon accounting) [62,63,64,65]. A complete LCA, together with a rigorous energy audit and catalyst lifetime studies, is identified as a necessary next step for the industrial validation of the PCSR route.

Concerning the overall energy and carbon footprint, it is instructive to benchmark the PCSR route against the main alternatives for hydrogen production. The thermal demand of the two-stage configuration is significant and, as discussed above, depends critically on the heat source: when the required heat is supplied by fossil fuels, the carbon footprint increases substantially, whereas the use of renewable heat or of the process co-products (biochar and non-condensable gas) as fuel markedly reduces net emissions. Direct gasification of MSW—the main competing thermochemical route—typically operates at 800–900 °C with reported energy efficiencies of 65–75%, but requires a gasifying agent (air, oxygen or steam) and extensive syngas cleaning to remove tars and particulates before downstream utilization [58]. Although gasification can achieve comparable hydrogen yields, it generally demands higher capital expenditure and a more complex gas-treatment train than the PCSR configuration, in which the catalytic bed converts tars and heavy hydrocarbons in situ. Compared with steam methane reforming (SMR), the incumbent industrial route, which emits 9–12 kg CO_2_-eq per kg H_2_ without carbon capture [57,59] and has a production cost of approximately USD 1.1–1.6 kg^−1^ [57], the PCSR route valorizes a renewable waste feedstock and avoids the fossil-derived emissions inherent to SMR. Recent life-cycle assessments confirm that waste-to-hydrogen routes reduce CO_2_-eq emissions by 50–69% relative to SMR, with MSW gasification exhibiting a global warming potential of approximately 5 kg CO_2_-eq·kg H_2_^−1^ [18]. Water electrolysis, in turn, is carbon-free only when powered by renewable electricity, requires 50–55 kWh of high-grade electricity per kg H_2_, does not valorize waste and remains more expensive than thermochemical routes in most markets [60]. Overall, the PCSR process offers a competitive trade-off among hydrogen yield, feedstock valorization and process simplicity, particularly when thermal integration and renewable heat sources are employed. A rigorous energy audit and a full life-cycle assessment at pilot scale are identified as necessary next steps to quantify these benefits definitively.

## 3. Materials and Methods

### 3.1. Municipal Solid Waste (MSW) Sampling and Preparation

The feedstock utilized in this study was sourced from the municipal solid waste (MSW) management systems of 32 municipalities located in the Serra Gaúcha region, Rio Grande do Sul, Brazil. Following the methodology described by Lazzarotto (2024) [23], the raw waste underwent a rigorous segregation and homogenization process to ensure representativeness. The samples were categorized into three distinct experimental groups: Fraction A (predominantly organic fraction), Fraction B (polymeric fraction consisting of plastics and synthetic fibers), and Fraction C (a standardized blend of organic and polymeric samples). To ensure kinetic reproducibility and minimize heat transfer limitations during the thermochemical conversion, all materials were comminuted through an individual grinding process using a knife mill, model DPM-2. The samples were stored at a temperature of −10 °C.

### 3.2. Feedstock Characterization

The physicochemical properties of the MSW fractions were determined through comprehensive analytical techniques. Proximate analysis (moisture, volatile matter, fixed carbon, and ash content) was performed according to ASTM D1762-84 [66]. Ultimate analysis (C, H, N, S) was conducted according to ASTM D5373-16 and ASTM D4239-18e1 [67,68], while the oxygen content was calculated by difference. The higher heating value (HHV) was measured using a bomb calorimeter, according to ASTM D5865-13 [69]. Chlorine content was determined by combustion in a calorimeter followed by the titrimetric method.

### 3.3. Pyrolysis and Catalytic Steam Reforming Experimental Setup

#### 3.3.1. Catalyst Characterization

**Figure 9:**
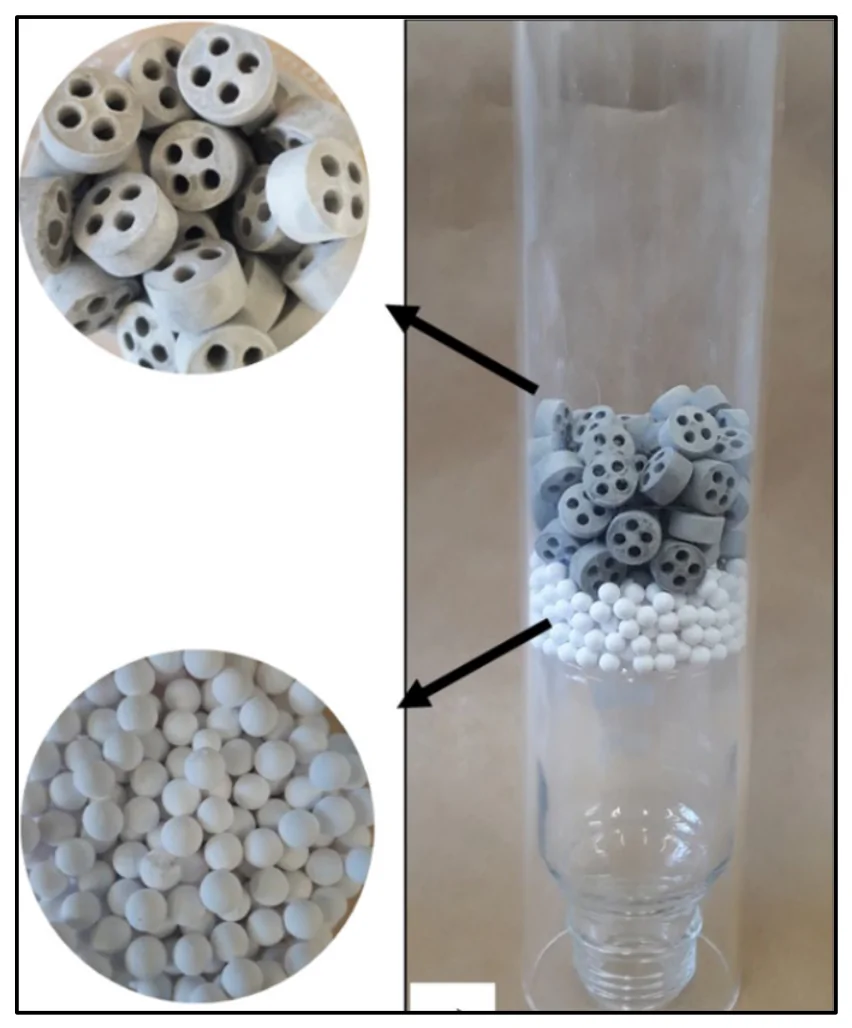
Catalytic bed used in the experiments.

A commercial Ni/Al_2_O_3_ catalyst was used in the PCSR experiments. Figure 9 illustrates the arrangement inside the catalytic reactor, highlighting the Ni catalyst (upper section) and the guard bed of alumina spheres (lower section). The Ni/Al_2_O_3_ catalyst used in the PCSR experiments was commercially sourced, with its characterization described by [70].

The catalyst employed in the PCSR process was characterized by N_2_ adsorption isotherm analysis, X-ray diffraction, and scanning electron microscopy. N_2_ adsorption/desorption analysis was performed using a Quantachrome Instruments analyzer, model NOVA 1200e (Quantachrome Instruments, Boynton Beach, FL, USA). Prior to analysis, the sample was degassed at 200 °C under vacuum. The specific surface area was determined by the BET method at 11 points, and the pore size distribution was obtained using the DFT method. The material exhibited a specific surface area of 10.3 m^2^·g^−1^. The adsorption isotherm was classified as Type IV, which is characteristic of mesoporous materials that often display hysteresis loops. The pore size distribution was concentrated in the mesoporous range (2–50 nm), with no evidence of micropores (<2 nm).

Scanning electron microscopy was performed using a Tescan microscope, model Mira 3 (TESCAN, Brno, Czech Republic). The structure consists of heterogeneously sized agglomerates with rounded contours, rich in micrometer-scale channels and covered by a rough, lighter layer, possibly associated with nickel deposition. Semi-quantitative elemental analysis of the catalyst was carried out by energy-dispersive X-ray spectroscopy, revealing O and Al as the major constituents, which were attributed to the catalyst support. The Ni content, corresponding to the active metal, was estimated at 9.8 wt.%. Significant concentrations of Ca and C were also observed, along with traces of Si. X-ray diffraction analysis was performed on a Shimadzu diffractometer, model XRD 6000. Two phases were identified in the catalyst support: alumina (Al_2_O_3_) and monocalcium dialuminate (CaAl_4_O_7_). Further catalyst characterization data are available in the Supplementary Materials.

The commercial Ni/Al_2_O_3_ catalyst employed in the PCSR experiments combines a Ni active phase (9.8 wt.%) dispersed on an alumina (Al_2_O_3_) support, with the latter also containing a monocalcium dialuminate (CaAl_4_O_7_) phase, as identified by XRD. In the reforming bed, nickel constitutes the active metal responsible for the dissociation of C–C and C–H bonds of hydrocarbons and oxygenates, whereas the alumina support provides thermal stability, mechanical resistance and a mesoporous structure (BET surface area of 10.3 m^2^·g^−1^, Type IV isotherm) that favors the dispersion of the active phase and the diffusion of reactants and products. The arrangement of the catalytic bed, with the Ni catalyst in the upper section and a guard bed of alumina spheres in the lower section, was designed to protect the active phase from deactivation by particulate matter and heavy tars carried by the pyrolysis volatiles. This catalyst configuration was previously characterized and validated for reforming applications, as described by Biondo et al. [53].

#### 3.3.2. PCSR and PSR Experiments

The MSW pyrolysis experiments were conducted in a horizontal tubular fixed-bed reactor made of AISI 310 stainless steel and manufactured by Fornos Sanchis (Porto Alegre, RS, Brazil), with a length of 980 mm and an inner diameter of 43 mm. The reactor was heated by two pairs of electrical resistances, each with a power output of 1900 W, both wrapped around the reaction tube. The temperature was set to 500 °C, and the reactor was held at this temperature for 1 h under a N_2_ flow of 100 mL·min^−1^ to ensure an inert atmosphere. Prior to the experiments, the samples were dried in an oven at 60 °C for 24 h to remove moisture. The condensable vapors and non-condensable gases were then directed to a second reactor (steam reforming reactor) connected in series.

The second reactor was a vertical tubular fixed-bed reactor, consisting of an AISI 310 stainless steel tube with an inner diameter of 25.4 mm and a length of 710 mm. The reactor was heated by a single electrical resistance with a power output of 4600 W, and the temperature was maintained at 900 °C. The steam reforming reactor was fed with steam at 150 °C at a mass flow rate of 0.5 kg·h^−1^. During the heating phase, a nitrogen flow was maintained to guarantee an inert atmosphere inside the reactor. After the temperature in the second reactor stabilized, steam feeding was initiated, and the experiment began by activating the first reactor.

**Figure 10:**
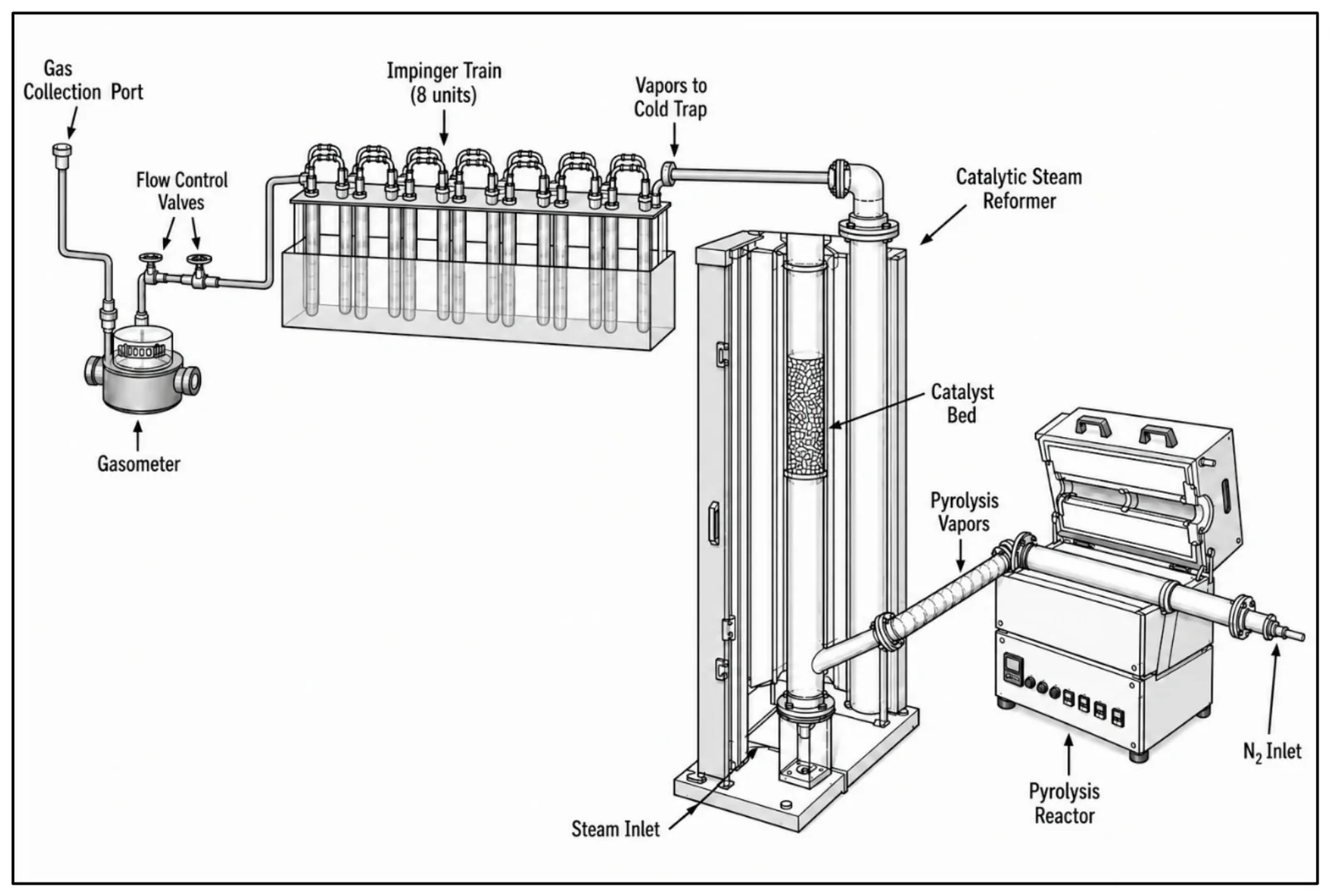
Schematic technical drawing of the PSRPCSR process.

The effluent stream from the reforming reactor was directed to a series of 10 bubblers connected in series, of which 5 contained 100 mL of isopropyl alcohol each, while the remaining 5 were left empty. After the residual condensable vapors were removed in the bubblers, the gas was sent to a gas meter and subsequently to an appropriate collection bag for further chromatographic analysis. The PSR process was conducted in the same way as the PCSR process, except that in the PSR process, no catalyst was placed in the second reactor. Figure 10 presents the schematic technical drawing of the process.

The key design and operating parameters of the two-stage reactor system are consolidated in Table 4. Both reactors operated at atmospheric pressure (≈1 atm). The pyrolysis reactor (horizontal tubular fixed-bed, AISI 310, 980 mm length, 43 mm internal diameter) was loaded with 30 g of the dried MSW sample per run and heated to 500 °C under a N_2_ flow of 100 mL·min^−1^, and it was held at this isothermal temperature for 60 min. The steam reforming reactor (vertical tubular fixed-bed, AISI 310, 710 mm length, 25.4 mm internal diameter) was charged with 150 g of the commercial Ni/Al_2_O_3_ catalyst—cylindrical tablets of 16.3 mm diameter and 11 mm height, 3 g each, with an apparent density of approximately 1.31 g·cm^−3^, corresponding to 50 tablets—forming a catalytic bed of approximately 378 mm height (calculated from the charged mass, tablet geometry, apparent density and reactor cross-section, assuming a packed-bed voidage of ε ≈ 0.40), and with 100 g of alumina spheres (bulk density ≈ 1.4 g·cm^−3^) as the guard bed, approximately 141 mm in height, placed below the catalytic section. The reforming reactor was maintained at 900 °C and fed with steam at 150 °C at a mass flow rate of 0.5 kg·h^−1^. Under these conditions, the gas residence time in the pyrolysis reactor was approximately 5.5 min (N_2_ flow expanded at 500 °C), and the contact time in the catalytic bed was approximately 0.3 s, corresponding to a gas hourly space velocity (GHSV) of approximately 1.3 × 10^4^ h^−1^. The effluent gas from the reforming reactor passed through a series of 10 bubblers and was collected in a gas sampling bag for chromatographic analysis.

The condensable vapors generated from MSW pyrolysis were subjected to separation into two phases, namely a light phase and a heavy phase. The separation was carried out in a rotary evaporator manufactured by Quimis, model Q344B1, at a temperature of 60 °C and an absolute internal pressure of 160 mmHg, for removal of the light phase. The heavy phase was then diluted in acetone (1:10) and sent for semi-quantitative compositional analysis in a gas chromatograph coupled to a mass spectrometer (GC-MS) HP series 6890/MSD 59736 (Agilent, Santa Clara, CA, USA).

The analysis of non-condensable gases (H_2_, CO, CH_4_, CO_2_) was performed using a gas chromatograph (Dani brand, Master GC model, DANI Instruments S.p.A., Cologno Monzese (MI), Italy) equipped with a thermal conductivity detector (TCD). Gas collection was initiated after the pyrolysis reactor reached the onset degradation temperature observed in the thermogravimetric analysis for each sample.

## 4. Conclusions

The comparative analysis of the three processes reveals a clear evolution toward gas yield. The PSR process showed average gas yields of 1.08, 1.89, and 0.97 Nm^3^·kgMSW^−1^ for fractions A, B, and C, respectively. The PCSR process showed average gas yields of 1.18, 1.54, and 1.5 Nm^3^·kgMSW^−1^ for fractions A, B, and C, respectively. Except for fraction B, the gas yield performance of PCSR was higher compared to that of PSR.

The comparative analysis of the three processes reveals a clear evolution toward hydrogen-rich gas production. The PSR process showed average hydrogen yields of 0.6, 1.1, and 0.85 Nm^3^·kgMSW^−1^ for fractions A, B, and C, respectively. The PCSR process showed average hydrogen yields of 1.1, 1.4, and 1.3 Nm^3^·kgMSW^−1^ for fractions A, B, and C, respectively. Thus, PCSR is more efficient in terms of hydrogen production yield.

The gas produced from PCSR exhibits higher purity in terms of H_2_ + CO_2_, with very low levels of contaminants (CO and CH_4_), which facilitates subsequent H_2_ separation and purification steps.

The quantitative evaluation of tar conversion over the Ni/Al_2_O_3_ catalyst was not performed in this study. Future investigations should include the gravimetric determination of the heavy (tar) phase collected downstream of the reforming reactor for each process configuration (e.g., by cold trapping and solvent impingement following an established tar sampling protocol, such as CEN/TS 15439 or equivalent), enabling the calculation of the tar conversion efficiency, $X_{tar}\left(\%\right)=\frac{m_{tar}^{PYR}-m_{tar}^{PCSR}}{m_{tar}^{PYR}}\times 100$, and the completion of the mass balance of the PCSR process.

In addition, the completion of the overall mass balance and of the carbon balance—including the gravimetric determination of the condensable (liquid) phase and the elemental analysis of the biochar—is required to establish the full carbon partitioning across the PYR, PSR, and PCSR stages and to validate the reported yields. The normalization of the H_2_ and gas yields to the catalyst mass or active Ni content is also suggested to facilitate a comparison with other catalytic systems.

Although the PSR process served as the thermal baseline, allowing the isolation of the catalytic contribution at 900 °C, a control experiment with an inert packed bed (e.g., bare SiC or quartz spheres) at the same reforming temperature is identified as a necessary next step to rigorously decouple thermal cracking from the catalytic effect under identical bed hydrodynamics. Such an experiment, together with the dedicated TOS tests, the spent-catalyst characterization and the tar conversion quantification, will complete the experimental framework required for the industrial validation of the PCSR processing of real municipal solid waste.

From a sustainability and industrial implementation perspective, the PCSR process offers a viable pathway for waste-to-hydrogen systems, aligning with circular economy principles. From an energetic standpoint, a first-order assessment indicates that the chemical energy carried by the hydrogen produced in the PCSR stage (≈11.9–15.1 MJ·kg^−1^ of MSW, based on the lower heating value of H_2_ of 10.8 MJ·Nm^−3^) exceeds the estimated thermal demand of the process—comprising the sensible heat of the feedstock, the vaporization of its moisture and the generation of steam (≈3.5–7.1 MJ·kg^−1^)—by a factor of approximately 2–3. This balance is further improved when the recoverable energy of the co-products is considered: in an industrial configuration, thermal integration through the combustion of the biochar (char yields of 22.6–29.8 wt.%), the recovery of heat from the hot product gas and the use of part of the non-condensable gas as fuel would substantially reduce the external thermal demand, offsetting the energy penalty associated with the high-temperature reforming stage at 900 °C. Moreover, the incremental hydrogen gain promoted by the catalytic stage (e.g., +0.50 Nm^3^·kg^−1^, corresponding to +83%, for fraction A) is achieved by converting tars and hydrocarbons that would otherwise be lost, so that the catalytic stage upgrades the feedstock rather than merely consuming additional energy. It must be emphasized, however, that this first-order balance excludes reactor heat losses and the electrical heating of the bench-scale unit, and that a rigorous energy audit at pilot scale, including measured power consumption and heat recovery, is required to confirm the net energetic viability of the PCSR route.

It must be acknowledged that the present study was conducted in batch mode over single runs; therefore, time-on-stream (TOS) data were not collected. Given that the reforming of MSW vapors is prone to carbon deposition (coking) over Ni-based catalysts, dedicated TOS experiments are required to demonstrate the operational stability and lifespan of the Ni/Al_2_O_3_ catalyst under the proposed PCSR conditions. Future investigations should therefore include continuous-operation tests, the quantification of carbon deposition by TGA and Raman spectroscopy, and the comprehensive characterization of the spent catalyst by XRD, SEM/TEM, and XPS in order to establish the deactivation kinetics, the long-term stability, and the regeneration strategies of the catalyst for the industrial upscaling of the PCSR processing of real municipal solid waste.

A thermodynamic equilibrium analysis of the catalytic steam reforming stage, based on the Gibbs free energy minimization method, is identified as a necessary next step to quantify how closely the experimental gas composition approaches the equilibrium distribution for each MSW fraction. Such an analysis would provide a quantitative basis for assessing process efficiency, catalyst performance and the remaining room for optimization of the operating variables (reforming temperature, steam-to-carbon ratio and residence time), supporting the industrial scale-up of the PCSR route.

Finally, it must be acknowledged that the present study characterized exclusively the fresh Ni/Al_2_O_3_ catalyst, and the analysis of the spent catalyst was not performed. Given that the reforming of MSW vapors is prone to carbon deposition over Ni-based catalysts, future investigations should include the comprehensive characterization of the spent catalyst to quantify and identify the nature of the deposited carbon. Specifically, thermogravimetric analysis (TGA) under an oxidizing atmosphere should be employed to quantify the total amount of deposited carbon (from the mass loss in the 400–700 °C region), while Raman spectroscopy should be used to discriminate between amorphous carbon (D-band, ~1350 cm^−1^) and graphitic carbon (G-band, ~1580 cm^−1^) as well as to determine their intensity ratio (I_D/I_G), which is indicative of the degree of graphitization. These analyses, together with XRD, SEM/TEM, and XPS, are essential to establish the deactivation kinetics, the long-term stability, and the regeneration strategies of the catalyst for the industrial upscaling of the PCSR processing of real municipal solid waste.

## Figures and Tables

**Table 1:** MSW proximate and ultimate analyses.

Parameter	Fraction A	Fraction B	Fraction C
Proximate analysis			
Moisture (g·100 g^−1^)	51.4 ± 2.2	6.5 ± 0.7	47.9 ± 3.6
Volatile Matter (g·100 g^−1^) ^1^	76.4 ± 2.0	84.4 ± 0.8	81.2 ± 0.8
Ash (g·100 g^−1^) ^1^	15.5 ± 1.3	7.0 ± 0.6	8.7 ± 0.9
Fixed Carbon (g·100 g^−1^) ^2^	8.1 ± 1.9	8.6 ± 0.2	10.0 ± 1.4
Ultimate analysis			
Carbon (g·100 g^−1^) ^1^	40.64	84.75	43.84
Hydrogen (g·100 g^−1^) ^1^	6.09	7.45	6.59
Nitrogen (g·100 g^−1^) ^1^	1.56	5.48	1.62
Sulfur (g·100 g^−1^) ^1^	0.16	0.09	0.16
Chlorine (g·100 g^−1^) ^1^	1.28 ± 0.09	1.18 ± 0.11	1.22 ± 0.07
HHV (MJ·kg^−1^) ^1^	17.84 ± 0.13	25.14 ± 2.89	19.03 ± 0.65

^1^ Dry basis. ^2^ Dry basis, obtained by difference.

**Table 2:** Gas yield and gas composition.

Fraction	Process	Gas Yield (Nm^3^·kg^−1^)	H_2_ (% vol.)	CO_2_ (% vol.)	CO (% vol.)	CH_4_ (% vol.)
A	PYR *	0.44	30.1	49.2	11.6	9.1
	PSR *	1.08	52.9	33.1	5.5	8.5
	PCSR *	1.18	38.1	59.5	0.5	1.9
B	PYR *	0.66	15.3	53.7	14.2	16.7
	PSR *	1.89	59.3	27.3	5.9	7.5
	PCSR *	1.54	32.2	65.8	0.5	1.5
C	PYR *	0.35	25.1	46.0	15.5	13.4
	PSR *	0.97	55.5	27.8	6.0	8.7
	PCSR *	1.50	39.9	56.2	0.5	3.4

* PYR: Pyrolysis. PSR: Pyrolysis and steam reforming. PCSR: Pyrolysis and catalytic steam reforming.

**Table 3:** Comparison of hydrogen production from waste-to-hydrogen studies via pyrolysis and catalytic steam reforming.

Feedstock	Catalyst	T_Reform (°C)	H_2_ Yield (Nm^3^·kg^−1^)	Gas Composition (H_2_ % vol.)	Ref.
Real MSW (fraction A)	Ni/Al_2_O_3_ (9.8% Ni)	900	1.10	38.1	This work
Real MSW (fraction B, polymeric)	Ni/Al_2_O_3_ (9.8% Ni)	900	1.40	32.2	This work
Real MSW (fraction C, mixed)	Ni/Al_2_O_3_ (9.8% Ni)	900	1.30	39.9	This work
MSW	Self-derived char-based (CaO:Fe:char)	850	2.02	71	[18]
HDPE (waste plastic)	Tire-derived ash	1000	1.86	50	[58]
Mixed waste plastics	Ni-Mg-Al	–	3.38	–	[58]
Polypropylene	Fe-activated carbon	–	2.52	–	[58]
Polypropylene	10% Ni/Al_2_O_3_	850	2.28	–	[59]

**Table 4:** Reactor configuration and operating conditions of the PYR/PSR/PCSR process.

Parameter	Pyrolysis (Reactor 1)	Steam Reforming (Reactor 2)
Type/material	Horizontal fixed-bed, AISI 310	Vertical fixed-bed, AISI 310
Length (mm)	980	710
Internal diameter (mm)	43	25.4
Temperature (°C)	500	900
Pressure	Atmospheric (≈1 atm)	Atmospheric (≈1 atm)
Sample mass (g)	30	30
Catalyst mass (g)	—	150 (Ni/Al_2_O_3_, 9.8% Ni)
Catalyst tablet	—	Ø 16.3 mm × 11 mm; 3 g; apparent density ≈ 1.31 g·cm^−3^
Catalytic bed height (mm)	—	≈378 (calculated, ε ≈ 0.40)
Guard bed mass (g)	—	100 (alumina spheres)
Guard bed height (mm)	—	≈141 (bulk density ≈ 1.4 g·cm^−3^)
Carrier gas	N_2_, 100 mL·min^−1^	N_2_, 100 mL·min^−1^
Steam feed	—	0.5 kg·h^−1^ at 150 °C
Isothermal reaction time	60 min	60 min
Residence/contact time	≈5.5 min (N_2_ at 500 °C, est.)	≈0.3 s (GHSV ≈ 1.3 × 10^4^ h^−1^, est.)

## Data Availability

Data can be made available upon reasonable request.

## Supplementary Materials

The following supporting information can be downloaded at: https://www.mdpi.com/article/10.3390/molecules31162917/s1, Figure S1. (a) Pieces of the studied catalyst and (b) schematic drawing with average dimensions of the pieces (dimensions in millimeters). Figure S2. (a) N_2_ adsorption/desorption isotherm and (b) pore size distribution by the DFT method of the commercial catalyst. Figure S3. SEM images of the commercial catalyst at magnifications of (a) 5000× and (b) 50,000×. Figure S4. Elemental distribution maps of the catalyst by energy dispersive spectroscopy. Figure S5. X-ray diffraction analysis of the studied catalyst. Table S1. Element concentration by EDS in the studied catalyst.
