# Supplementary material for: Hydrogen-Rich Gas Production from Municipal Solid Waste via Integrated Pyrolysis and Catalytic Steam Reforming over Ni/Al2O3 Catalyst
Source: Molecules. 2026 Aug 20;31(16):2917. doi: 10.3390/molecules31162917 (PMC13515945; doi:10.3390/molecules31162917)
Supplement: Supplementary file 1 [file molecules-31-02917-s001.zip › molecules-4503252-supplementary.pdf]

Supplementary Material: Detailed Catalyst Characterization

Hydrogen-Rich Gas Production from Municipal Solid Waste via Integrated Pyrolysis and Catalytic Steam Reforming over Ni/Al_2O_3 Catalyst

S1. Catalyst Characterization

The catalyst is shown in **Figure S1a**. Each piece has an approximate mass of **3.0 g** and dimensions as shown in **Figure S1b**. For catalyst characterization, 5 pieces were randomly selected, ground, homogenized, and the material was sent for analysis. The catalyst was characterized by N_2 adsorption isotherm, X-ray diffraction, and scanning electron microscopy. The results of these analyses are presented and discussed in this section.

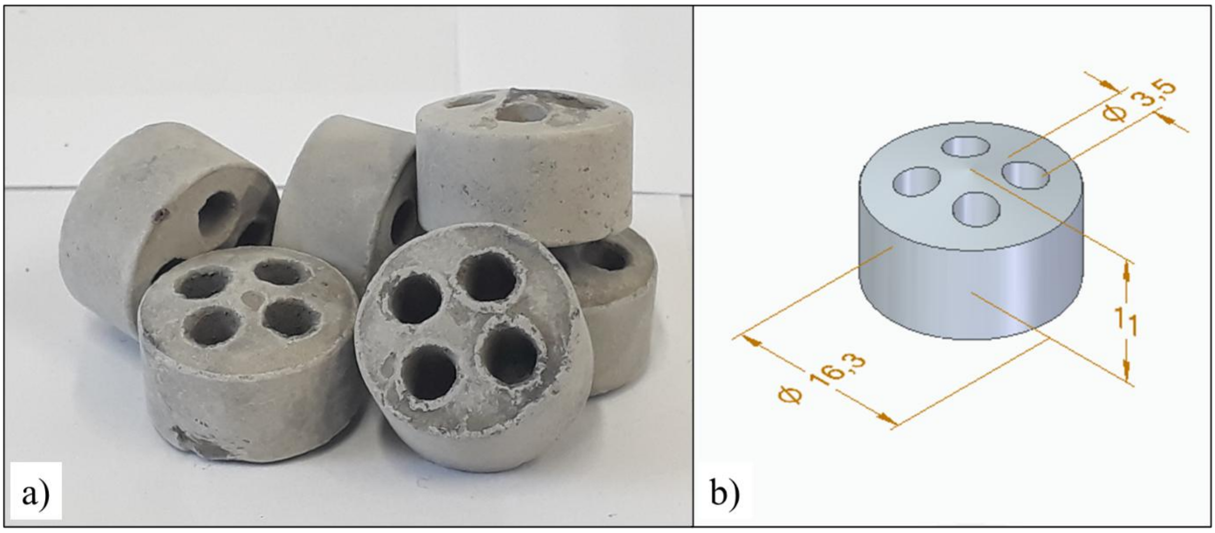

Figure S1. (a) Pieces of the studied catalyst and (b) schematic drawing with average dimensions of the pieces (dimensions in millimeters).

S1.1. N_2 Adsorption/Desorption Isotherm

N_2 adsorption/desorption analysis was performed on a Quantachrome Instruments analyzer, model NOVA 1200e. Prior to analysis, the sample was degassed at **200 °C** under vacuum. The surface area was calculated by the BET method at 11 points, and the pore size distribution was obtained using the DFT method. The material exhibited a specific surface area of **10.3 $m^2 \cdot g^{-1}$**. The adsorption isotherm, presented in **Figure S2a**, is classified as Type IV according to the IUPAC classification. Type IV isotherms are characteristic of mesoporous materials and frequently exhibit hysteresis loops, such as the Type H3 hysteresis observed in **Figure S2a**.

The pore size distribution is shown in **Figure S2b**. The pores are concentrated in the mesoporous region (2–50 nm), and the presence of micropores (<2 nm) is not observed. These larger-diameter pores allow the diffusion of molecules to the active sites of the catalyst.

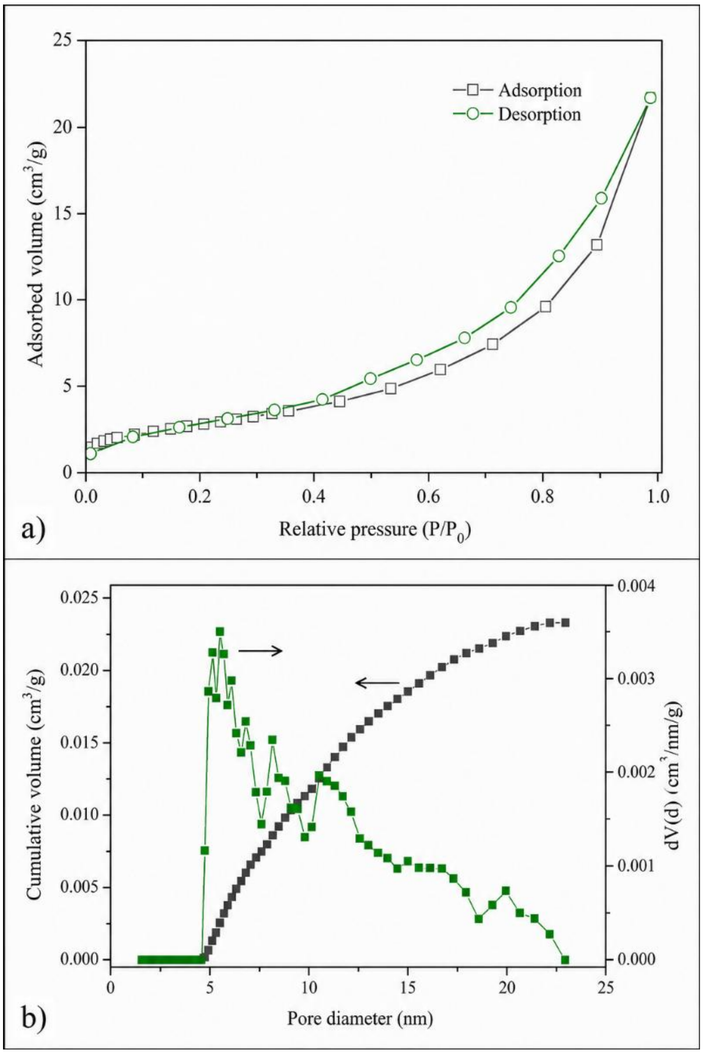

Figure S2. (a) N_2 adsorption/desorption isotherm and (b) pore size distribution by the DFT method of the commercial catalyst.

S1.2. Scanning Electron Microscopy and Energy Dispersive Spectroscopy

Scanning electron microscopy was performed on a Tescan microscope, model Mira 3. The micrographs are presented in **Figure S3**. A structure composed of heterogeneously sized agglomerates with rounded contours is observed. The structure is rich in micrometer-scale channels. In **Figure S3b**, at higher magnification, the structure is observed to be covered by a rough, lighter layer, possibly related to nickel deposition.

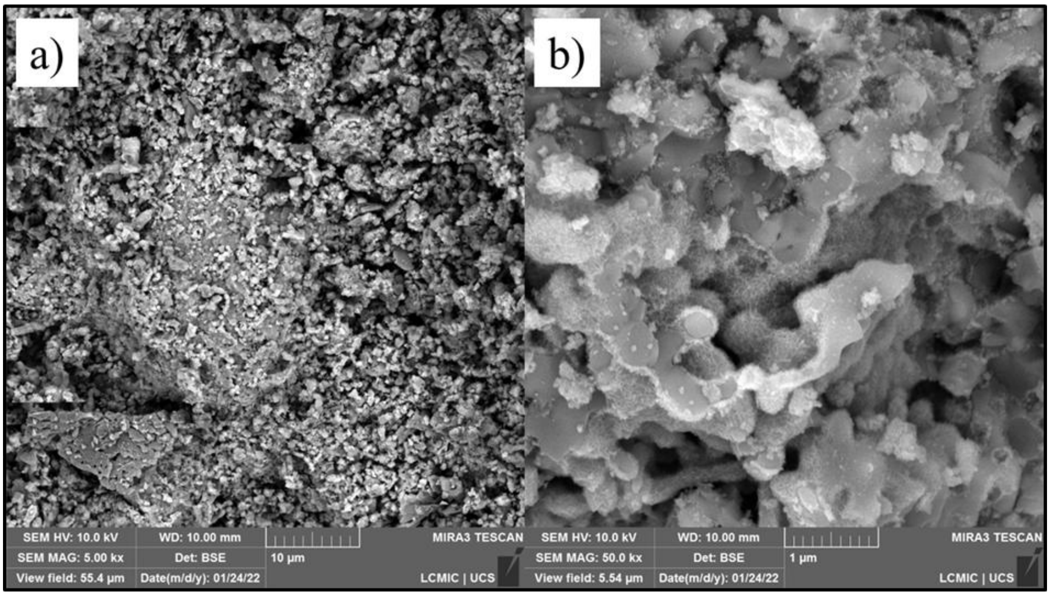

Figure S3. SEM images of the commercial catalyst at magnifications of (a) 5000× and (b) 50,000×.

A semi-quantitative analysis of the elemental composition of the catalyst was performed by energy dispersive spectroscopy. The results are presented in **Table S1**. The major participation of O and Al is observed, associated with the catalyst support. The Ni concentration, the active metal of the catalyst, was estimated at **9.8 wt.%**. Significant concentrations of Ca and C are also noteworthy, along with traces of Si.

Table S1. Element concentration by EDS in the studied catalyst.

Element	Concentration (wt.%)
O	40.0
Al	35.1
Ni	9.8
Ca	9.2
C	5.7
Si	0.2

The elemental distribution maps are presented in **Figure S4**. A homogeneous distribution of Ni is observed at the evaluated scale, as well as for the other elements composing the catalyst support. C and Si exhibited a more heterogeneous distribution, with points of higher concentration.

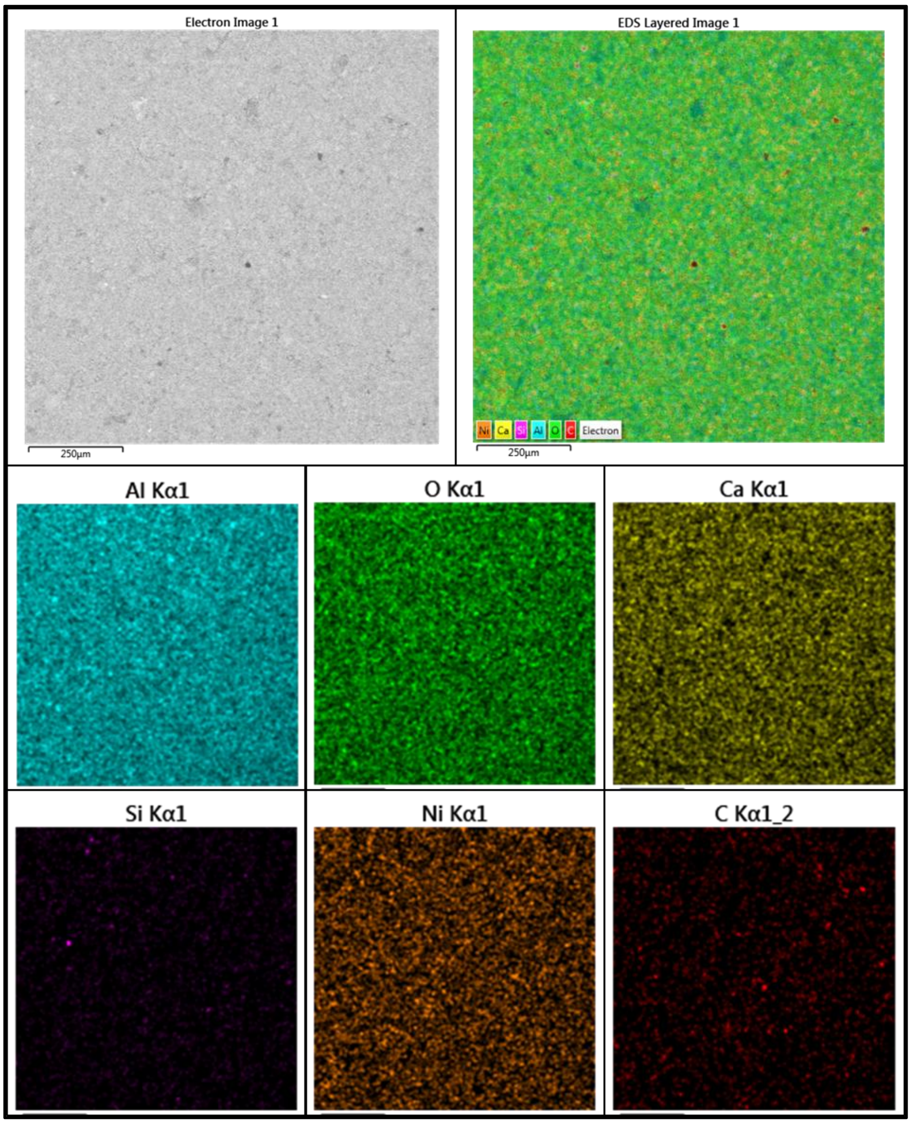

Figure S4. Elemental distribution maps of the catalyst by energy dispersive spectroscopy.

S1.3. X-Ray Diffraction

The application of X-ray diffraction to catalysts aims to identify the support phases and also the oxidation state of the active metal. The analysis was performed on a Shimadzu diffractometer, model XRD 6000. The diffraction pattern is presented in **Figure S5**. Two phases composing the catalyst support were identified: alumina (Al_2O_3) and monocalcium dialuminate ($CaAl_4O_7$). The identified phases corroborate the Al, O, and Ca contents presented in **Table S1**. Due to the overlap of the intense peaks of the support phases with the peaks referring to Ni and NiO, it was not possible to identify the oxidation state of nickel in the catalyst.

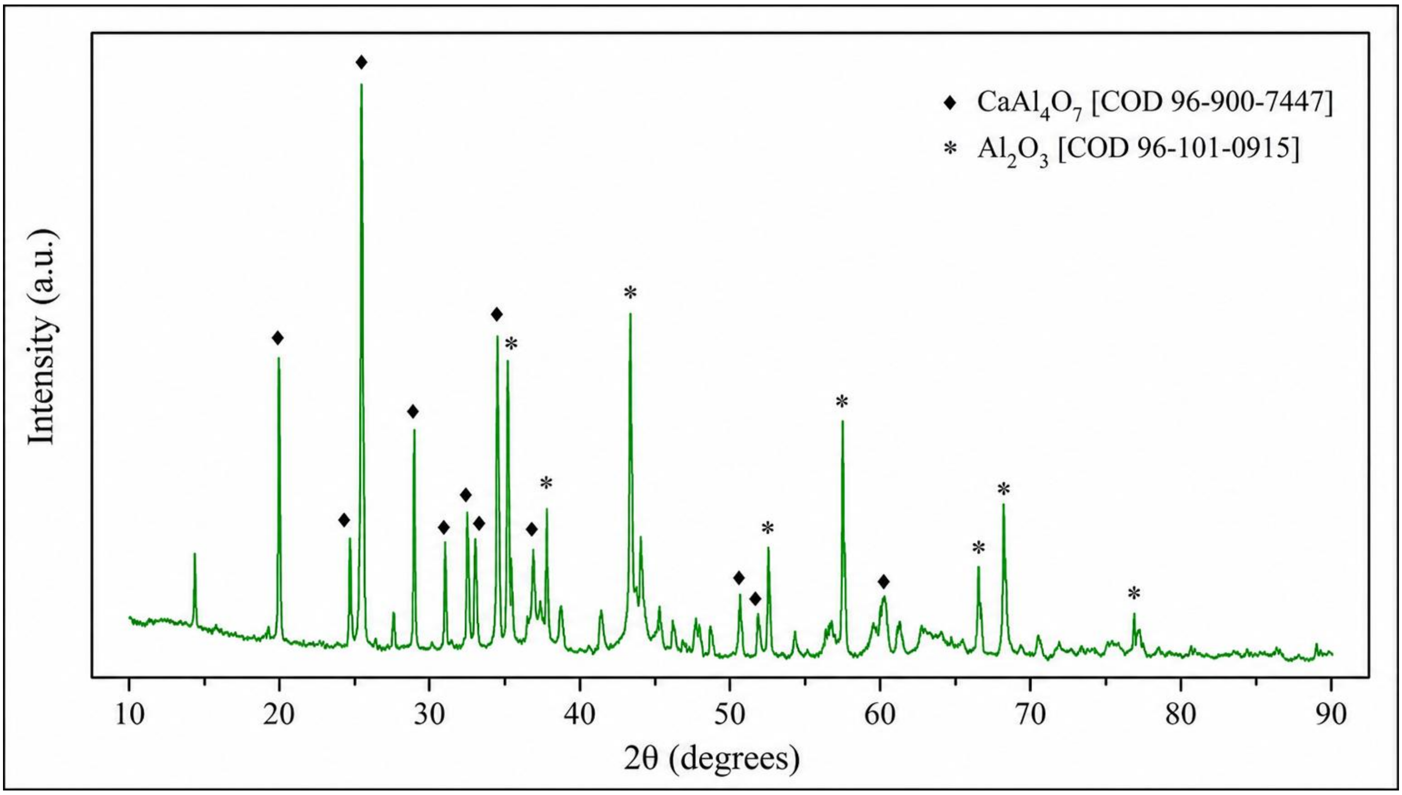

Figure S5. X-ray diffraction analysis of the studied catalyst.
